# Large-Scale Gene-Centric Analysis Identifies Novel Variants for Coronary Artery Disease

**DOI:** 10.1371/journal.pgen.1002260

**Published:** 2011-09-22

**Authors:** The IBC 50K CAD Consortium

**Affiliations:** Queensland Institute of Medical Research, Australia

## Abstract

Coronary artery disease (CAD) has a significant genetic contribution that is incompletely characterized. To complement genome-wide association (GWA) studies, we conducted a large and systematic candidate gene study of CAD susceptibility, including analysis of many uncommon and functional variants. We examined 49,094 genetic variants in ∼2,100 genes of cardiovascular relevance, using a customised gene array in 15,596 CAD cases and 34,992 controls (11,202 cases and 30,733 controls of European descent; 4,394 cases and 4,259 controls of South Asian origin). We attempted to replicate putative novel associations in an additional 17,121 CAD cases and 40,473 controls. Potential mechanisms through which the novel variants could affect CAD risk were explored through association tests with vascular risk factors and gene expression. We confirmed associations of several previously known CAD susceptibility loci (eg, 9p21.3:p<10^−33^; *LPA*:p<10^−19^; 1p13.3:p<10^−17^) as well as three recently discovered loci (*COL4A1/COL4A2*, *ZC3HC1*, *CYP17A1*:p<5×10^−7^). However, we found essentially null results for most previously suggested CAD candidate genes. In our replication study of 24 promising common variants, we identified novel associations of variants in or near *LIPA*, *IL5*, *TRIB1*, and *ABCG5/ABCG8*, with per-allele odds ratios for CAD risk with each of the novel variants ranging from 1.06–1.09. Associations with variants at *LIPA*, *TRIB1*, and *ABCG5/ABCG8* were supported by gene expression data or effects on lipid levels. Apart from the previously reported variants in *LPA*, none of the other ∼4,500 low frequency and functional variants showed a strong effect. Associations in South Asians did not differ appreciably from those in Europeans, except for 9p21.3 (per-allele odds ratio: 1.14 versus 1.27 respectively; P for heterogeneity = 0.003). This large-scale gene-centric analysis has identified several novel genes for CAD that relate to diverse biochemical and cellular functions and clarified the literature with regard to many previously suggested genes.

## Author Summary

Coronary artery disease (CAD) has a strong genetic basis that remains poorly characterised. Using a custom-designed array, we tested the association with CAD of almost 50,000 common and low frequency variants in ∼2,000 genes of known or suspected cardiovascular relevance. We genotyped the array in 15,596 CAD cases and 34,992 controls (11,202 cases and 30,733 controls of European descent; 4,394 cases and 4,259 controls of South Asian origin) and attempted to replicate putative novel associations in an additional 17,121 CAD cases and 40,473 controls. We report the novel association of variants in or near four genes with CAD and in additional studies identify potential mechanisms by which some of these novel variants affect CAD risk. Interestingly, we found that these variants, as well as the majority of previously reported CAD variants, have similar associations in Europeans and South Asians. Contrary to prior expectations, many previously suggested candidate genes did not show evidence of any effect on CAD risk, and neither did we identify any novel low frequency alleles with strong effects amongst the genes tested. Discovery of novel genes associated with heart disease may help to further understand the aetiology of cardiovascular disease and identify new targets for therapeutic interventions.

## Introduction

Coronary artery disease (CAD) has a substantial genetic component which is incompletely characterised. Genomewide association (GWA) studies have recently identified several novel susceptibility loci for CAD [1]–[9]. Because GWA studies involve assumption-free surveys of common genetic variation across the genome, they can identify genetic regions responsible for previously unsuspected or unknown disease mechanisms. However, despite the success of the GWA approach, it has potential limitations. Because CAD loci identified through GWA studies have predominantly been found in regions of uncertain biological relevance, further work is required to determine their precise contribution to disease aetiology. Furthermore, in contrast with their high coverage of common genetic variation, GWA studies tend to provide limited coverage of genes with well-characterised biological relevance (“candidate genes”) [2], particularly in relation to lower frequency genetic variants (such as those with minor allele frequencies of 1–5%). Such variants are also often difficult to impute from GWA data. Although candidate gene studies should provide more comprehensive coverage of lower frequency and functional variants than GWA studies, most have been inadequately powered.

To complement GWA studies, we undertook a large-scale gene-centric analysis of CAD using a customised gene array enriched with common and low frequency variants in ∼2,100 candidate cardiovascular genes reflecting a wide variety of biological pathways [10]. The array's potential to identify disease-associated lower frequency variants has been demonstrated by previous identification of strong independent associations with 2 variants in the *LPA* gene - rs3798220 (minor allele frequency 2%), and rs10455872 (7%) - and CAD risk [11]. We have now investigated this gene array in a further 13 studies comprising a total of 15,596 CAD cases and 34,992 controls. To enable interethnic comparisons, participants included 4,394 cases and 4,259 controls of South Asian descent, an ethnic group with high susceptibility to CAD. For further evaluation of putative novel associations, we attempted to replicate them in an additional 17,121 cases and 40,473 controls.

## Results

**Figure 1:**
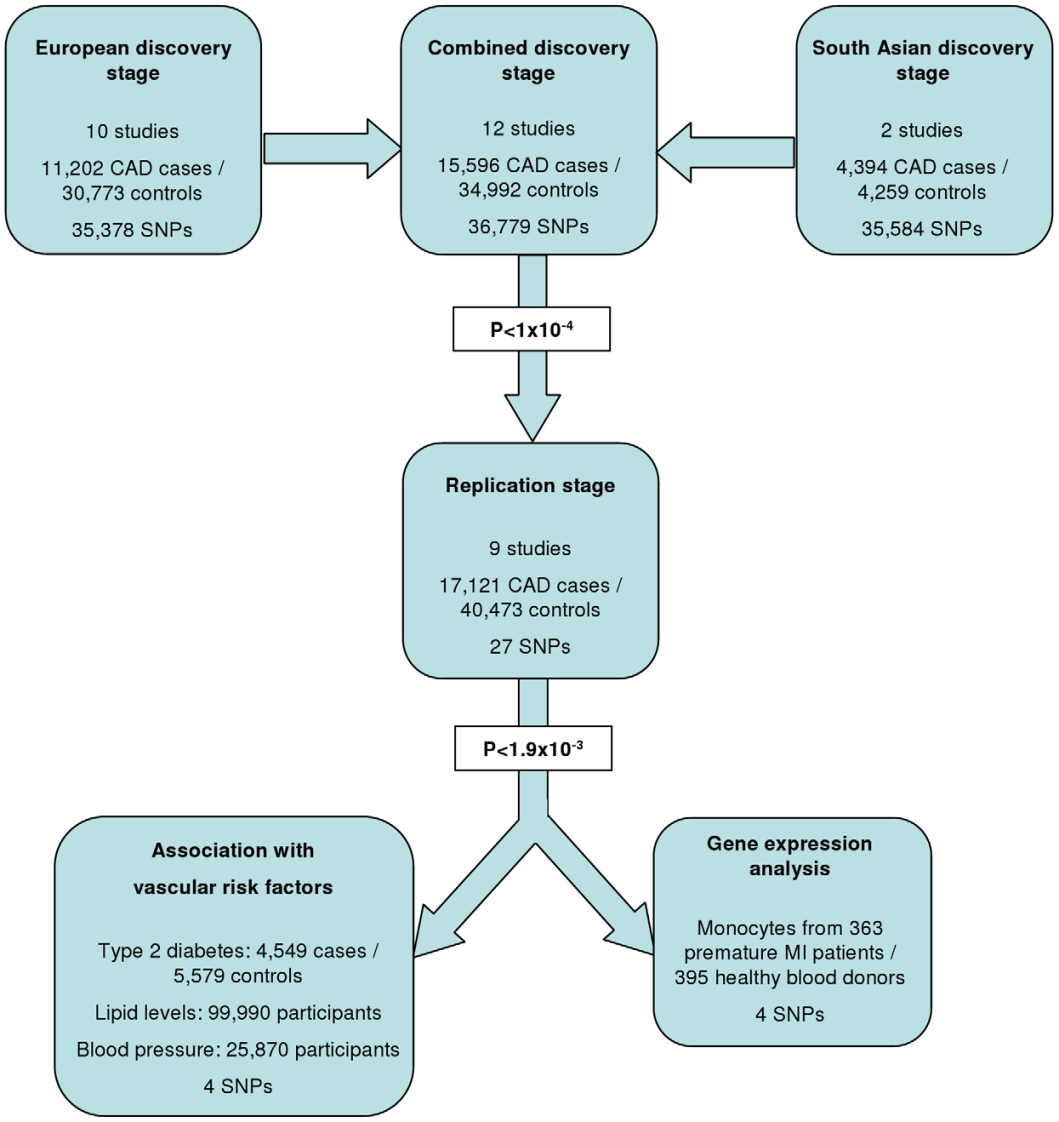
Design of the study.

The experimental strategy used is shown in Figure 1. In the discovery phase we genotyped participants from 12 association studies of CAD/myocardial infarction (MI), including a total of 11,202 cases and 30,733 controls of European descent (10 studies), plus 4,394 South Asian cases and 4,259 South Asian controls (2 studies) (Table 1, Table S1, with further details of the studies given in Text S1).

**Table 1:** Summary details of discovery and replication stage studies.

Stage	Study	Cases / Controls	Male (%)	Mean age (SD) of cases at diagnosis	Number of cases with MI (%)	Version of IBC array**
European discovery	ARIC	424 / 8447	46.4	-°	368 (82.7)	V2
	BHF-FHS	2101 / 2426	63.3	49.8 (7.7)	1538 (73.2)	V1
	BLOODOMICS - Dutch	1462 / 1222	72.6	48.8 (12.0)	1462 (100)	V2
	BLOODOMICS - German	1910 / 1932	63.3	59.2 (10.9)	1181 (61.8)	V2
	CARDIA	87 / 1343	46.8	-°	86 (100)	V2
	CHS	737 / 3155	43.9	-°	381 (50.5)	V2
	FOS	59 / 6976	45.1	-°	13 (22.0)	V2
	MONICA-KORA	275 / 1413	57.5	52.9 (9.4)	>50%*	V1
	PennCATH	1027 / 489	66.0	54.2 (8.8)	439 (40.6)	V1
	PROCARDIS	3120 / 3330	59.2	61.0 (8.7)	2136 (68.5)	V2
	Total	11,202 / 30,733				
South Asian discovery	PROMIS	1856 / 1905	82.5	53.3 (10.7)	1856 (100)	V1
	LOLIPOP	2538 / 2354	83.7	-	1125 (44.4)	V2
	Total	4394 / 4259				
Replication	CARDIoGRAM†	15,949 / 38,823	57.0	53.5 (9.8)	10,890 (68.3)	N/A
	EPIC-NL	1172 / 1650	30.6	51.9 (10.6)	341 (30.3)	V3
	Total	17,121 / 40,473				

ARIC = Atherosclerosis Risk In Communities; BHF-FHS = British Heart Foundation Family Heart Study; CARDIA = Coronary Artery Risk Development in Young Adults; CHS = Cardiovascular Health Study; FOS = Framingham Offspring Study; LOLIPOP = London Life Sciences Prospective Population Cohort; PROCARDIS = Precocious Coronary Artery Disease; PROMIS = Pakistan Risk of Myocardial Infarction Study; EPIC-NL = European Prospective Investigation into Cancer & Nutrition (Netherlands) cohort.

*All MONICA-KORA cases are either MI or sudden cardiac death.

**V2 contains an additional 132 genes (3,857 SNPs) compared to V1. SNPs on V2 were only analysed in studies that used the V2 array.

**†:** Details of studies in the CARDIoGRAM Consortium are presented in Table S6.

**°:** The 4 studies in the CARe Consortium contributed data only on prevalent CAD cases at baseline for whom ages were not available.

### Associations with known CAD loci

**Figure 2:**
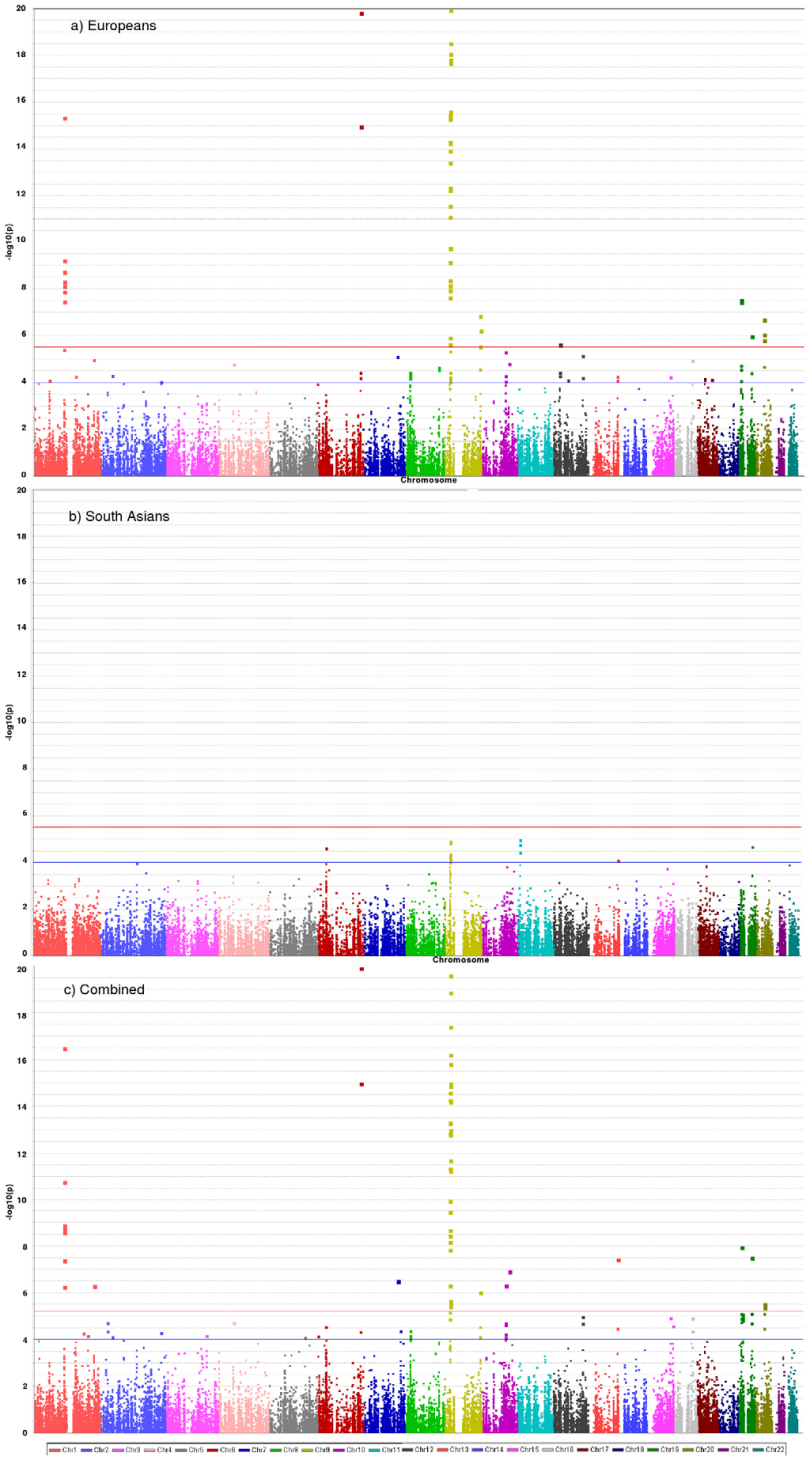
Manhattan plots for discovery stage meta-analyses. Y-axis shows unadjusted −log_10_(P values) from fixed-effect meta-analysis of discovery stage studies. NB: European and Combined plots are truncated at P = 10^−20^. Blue horizontal line at P = 10^−4^ indicates threshold for replication; Red horizontal line at P = 3×10^−6^ indicates array-wide significance level.

36,799 SNPs passed QC and frequency checks and were included in the meta-analysis (reasons for exclusion of variants in each study are given in Table S2). The distribution of association P values in the discovery stage analyses are shown in Figure 2. We found significant associations with CAD for several previous GWA-identified loci contained on the array including 9p21.3 (rs1333042, combined European and South Asian P = 1.1×10^−37^) and 1p13.3 (rs646776, 3.1×10^−17^; Table S3). We also confirmed associations of other genes with strong prior evidence including the first association of a variant at the apolipoprotein E locus at genomewide significance (*APOE/TOMM40*, rs2075650, P = 3.2×10^−8^), as well as associations at apolipoprotein (a) (*LPA*, rs10455872, P = 1.2×10^−20^), and low density lipoprotein receptor (*LDLR*, rs6511720, P = 1.1×10^−8^; Table S3). However, we found no persuasive evidence of association of several prominently-studied genes and variants for which the previous epidemiological evidence has been inconclusive, even though the majority of these loci were well-tagged (Table S4) and the current study was well-powered to detect associations of modest effect (Figure S1). Notable variants that did not show significant association included the angiotensin converting enzyme (*ACE*) insertion/deletion polymorphism, the cholesteryl-ester transfer protein (*CETP*) *Taq*1B polymorphism and the paraoxonase 1 (*PON1*) Q192R polymorphism (Table S4). Perhaps contrary to expectation, apart from the *LPA* variant rs3798220, we did not observe any other strong association (odds ratio >1.5) among the ∼4,500 low frequency (1–5%) variants and/or variants with suspected or known functional impact on protein structure/function or gene expression specifically selected for the inclusion on the array (Table S3).

### Novel CAD loci

**Figure 3:**
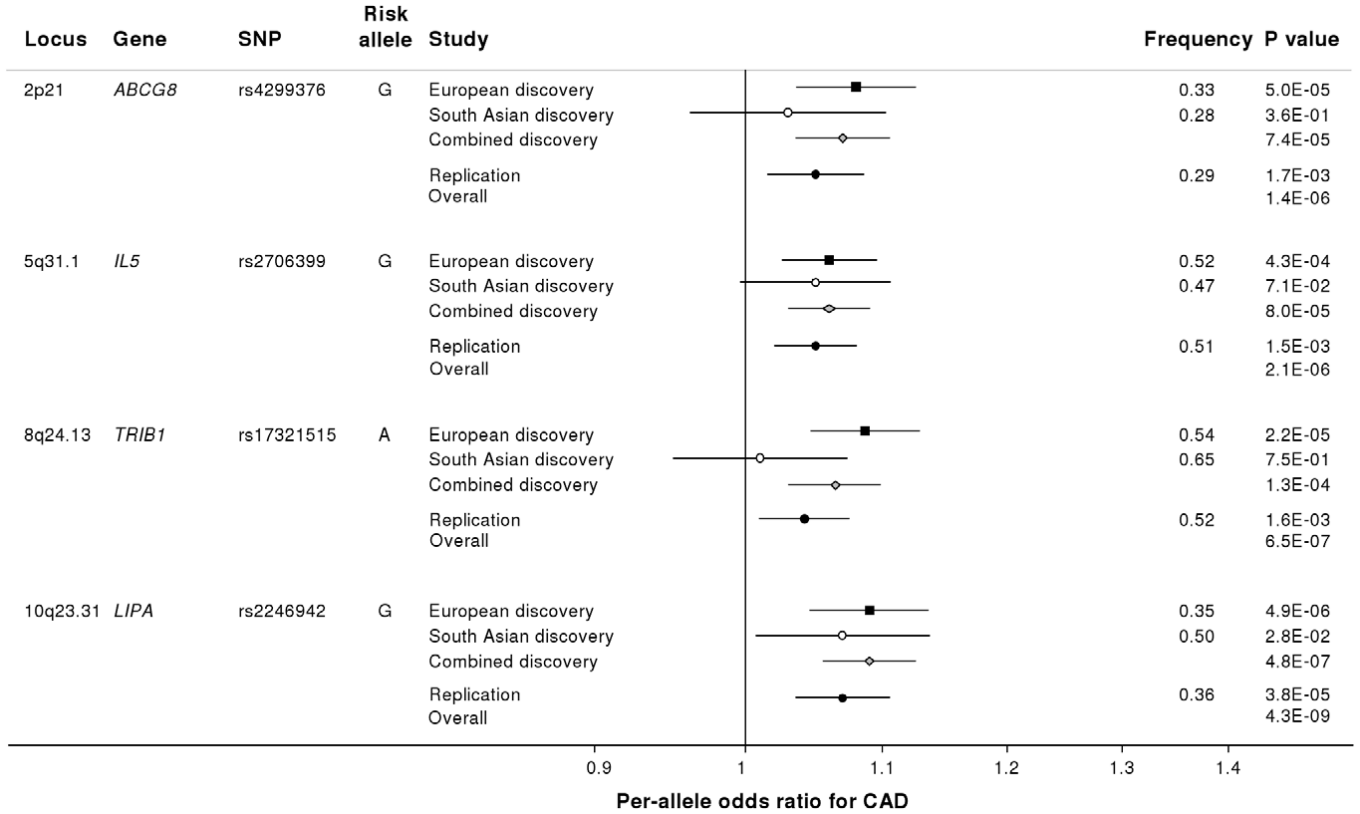
Novel loci identified in the current study. Loci ordered by chromosomal position. SNP = SNP showing strongest evidence of association in discovery stage studies; Frequency = pooled frequency of risk allele across controls; European discovery = per-allele odds ratio, confidence interval and 2-tailed P value from fixed-effect meta-analysis of European discovery stage studies; South Asian discovery = per-allele odds ratio, confidence interval and 2-tailed P value from fixed-effect meta-analysis of South Asian discovery stage studies; Combined discovery = per-allele odds ratio, confidence interval and 2-tailed P value from fixed-effect meta-analysis of all European and South Asian discovery stage studies combined; Replication = per-allele odds ratio, confidence interval and 1-tailed P value from fixed-effect meta-analysis of replication stage studies comprising non-overlapping participants from CARDIoGRAM plus all participants from EPIC-NL; Overall = P value from relevant discovery stage studies combined with the replication stage P value using Fisher's method.

**Figure 4:**
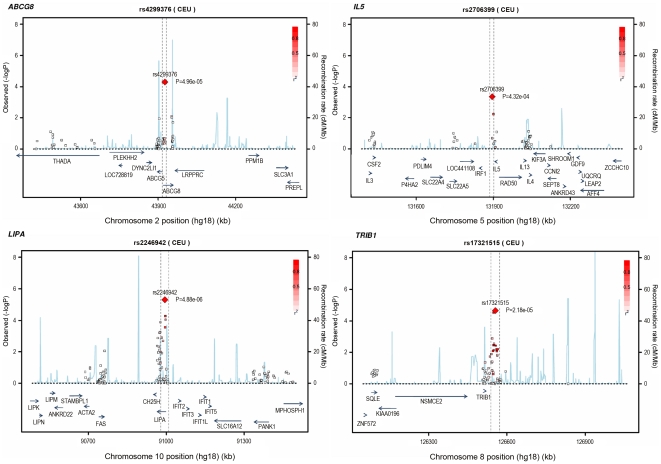
Regional association plots for novel loci identified. All SNPs included in meta-analysis of the European discovery stage studies are represented by diamonds, with the lead SNP (lowest P value) at each locus represented by a large red diamond. Genes are represented as horizontal arrows, with the direction of the arrow reflecting the direction of transcription. Recombination rates are represented as vertical blue peaks based on the Hapmap 2 CEU population. P values are from fixed-effect meta-analysis. LD, represented as r^2^, is estimated using the controls from the BHF-FHS study, or Hapmap 2 CEU population where data were not available in BHF-FHS. Vertical dashed lines represent the extent of LD with the lead SNP, based on an r^2^ threshold of 0.5 in the Hapmap 2 CEU population. The genes between these lines represent the most likely candidate genes for each association signal.

Based on simulations conducted prior to the analysis (Figure S2), loci were eligible for replication if unadjusted P-values for CAD were <1×10^−4^ in either the primary (each ethnic group analysed separately) or secondary (combined) analyses and the loci had not been previously established with CAD. This identified 27 loci in total: 15 in the European only analysis, 3 in the South Asian only analysis, and 9 in the combined analysis (Table S5). A recent GWA meta-analysis from the CARDIoGRAM Consortium with some overlapping cohorts to those in our study, reports discovery of three of these loci [12]: *COL4A1/COL4A2*, *ZC3HC1*, *CYP17A1*. The P values observed for the lead variants at these loci in the current study were: *COL4A1/COL4A2*: rs4773144, P = 3.5×10^−8^; *ZC3HC1*: rs11556924, P = 3.1×10^−7^; *CYP17A1*: rs3824755, P = 1.2×10^−7^, providing further strong evidence for the association of these loci with CAD. Hence, only the lead SNPs at the 24 remaining loci were taken forward for replication. This was done *in silico* in 17,121 CAD cases and 40,473 controls, all of whom were of white European ancestry and derived from non-overlapping cohorts from CARDIoGRAM and EPIC-NL (Text S1, Table S6). The power of our replication sample to confirm significant associations is shown in Figure S1. Of the 24 variants taken forward, four were independently replicated (1-tailed Bonferroni-corrected P<0.05 is P<1.9×10^−3^; Figure 3, Table S5), comprising variants in or adjacent to: *LIPA*, *IL5*, *TRIB1* and *ABCG5/ABCG8* (Figure 4). For the variant at the *LIPA* locus, the combined P-value was 4.3×10^−9^, exceeding conventional thresholds for GWA studies. For each of the *IL5*, *TRIB1* and *ABCG5/ABCG8* variants, the P-value was <3×10^−6^, exceeding array-wide levels of significance (Figure 3). CAD associations in the individual component studies are shown in Figure S3. The CAD associations for these loci did not vary materially by age, sex or when restricted to the MI subphenotype (Figure S4).

### Potential mechanisms

**Figure 5:**
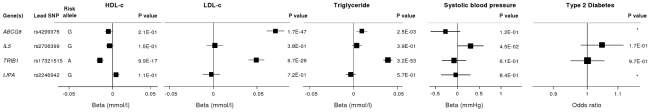
Effects of novel CAD loci on known cardiovascular risk factors. HDL-c = high-density lipoprotein cholesterol; LDL-c = low-density lipoprotein cholesterol; Beta/odds ratio = combined effect from meta-analysis of SNP versus blood pressure/lipids/T2D. Results for lipids from meta-analysis of 46 GWA studies containing up to 99,900 individuals [15]. Results for blood pressure from the Global BPGen Consortium: a meta-analysis of 17 GWA studies containing 25,870 individuals [14]. Results for diabetes from the DIAGRAM Consortium: a meta-analysis of 3 GWA studies containing 4,549 T2DM cases and 5,579 controls [13]. * No results available due to poor quality of SNP imputation.

**Figure 6:**
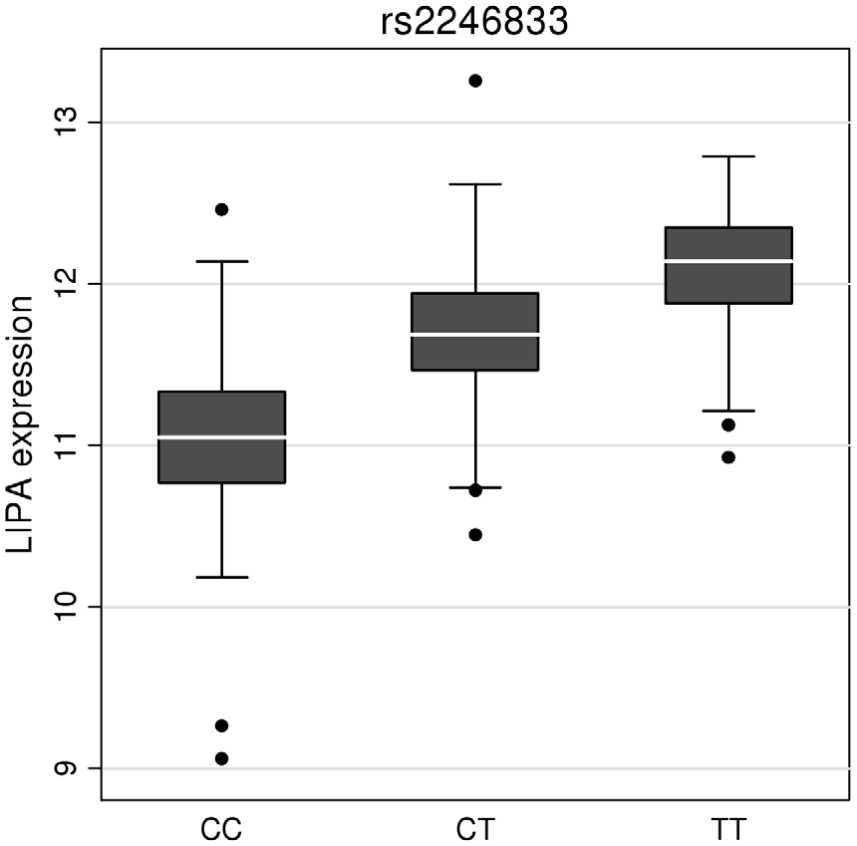
Evidence for an eQTL association in the *LIPA* gene. Expression levels of *LIPA* in monocytes taken from 758 individuals assembled by the Cardiogenics Consortium partitioned by genotype of SNP rs2246833. Boxes indicate interquartile ranges with a white horizontal line indicating the median. Error bars represent absolute minimum and maximum levels with dots showing those levels considered to be outliers. rs2246833 is in strong linkage disequilibrium (r^2^ = 0.93; D′ = 1) with the CAD-associated variant at the *LIPA* locus (rs2246942). The T allele, which is associated with increased *LIPA* expression, is inherited with the G allele of rs2246942, which is associated with increased risk of coronary disease.

To investigate whether the 4 newly identified loci associate with cardiovascular risk traits, we interrogated available data from previous GWA meta-analyses of diabetes mellitus (n = 10,128 individuals) [13], systolic blood pressure (n = 25,870) [14], and low-density (LDL) and high-density (HDL) lipoprotein-cholesterol and triglycerides (n = 99,900) [15]. This showed that the risk allele at the *TRIB1* locus was associated with higher triglyceride (P = 3.2×10^−53^), higher LDL-C (P = 6.7×10^−29^) and lower HDL-C (P = 9.9×10^−17^) and that the *ABCG5/ABCG8* risk allele was associated with higher LDL-C (P = 1.7×10^−47^; Figure 5). We also examined the association of the novel risk variants with gene expression in full transcriptomic profiles of circulating monocytes derived from 363 patients with premature myocardial infarction and 395 healthy blood donors from the *Cardiogenics* study (Text S1). We found a highly significant association (P = 1.0×10^−124^) of the risk allele at the *LIPA* locus with LIPA mRNA levels in these cells explaining ∼50% of the variance in the expression of the gene (Figure 6).There were no other highly significant associations between CAD risk alleles and gene expression at the novel loci (Table S7a and S7b).

### Ethnic-specific analyses

We explored whether associations of loci with CAD differed between individuals of white European ancestry and South Asian ancestry. For most loci, frequency of risk alleles and pattern of risk associations did not differ qualitatively by ethnicity, although the evidence of association was often weaker in South Asians, perhaps due to lower power (Figure 3, Tables S3 and S5). For the 9p21.3 locus, despite similar risk allele frequencies (Table S3), odds ratios were higher in Europeans than South Asians (rs1333042: 1.27 v 1.14; P = 0.003 for difference), though common haplotype frequencies did not vary by ethnicity (Table S8). The three variants at the *TUB*, *LCT* and *MICB* loci selected for replication on the basis of South Asian-specific results did not show evidence of association in Europeans (Table S5).

## Discussion

Our in-depth study of ∼2,100 candidate genes has yielded several novel and potentially important findings, adding to the emerging knowledge on the genetic determination of CAD. First, we have identified several novel genes for CAD. These genes relate to diverse biochemical and cellular functions: *LIPA* for the locus on 10q23.3; *IL5* (5q31.1); *ABCG5/ABCG8* (2p21); *TRIB1* (8q24.13); *COL4A1/COL4A2* (13q34); *Z3HC1* (7q32.3); and *CYP17A1* (10q24.3). We have furnished evidence directly implicating the candidacy of these genes, either because the locations of the signals discovered are within a narrow window of linkage disequilibrium or because there is evidence of a mechanistic effect, or both. Second, we have provided large-scale refutation of the relevance of many prominent candidate gene hypotheses in CAD, thereby clarifying the literature. Third, contrary to expectation, we did not observe highly significant novel associations between low frequency variants and CAD risk, despite study of >4,500 such variants. Fourth, we have confirmed the relevance of several previously established CAD genes to both Europeans and South Asians, without finding qualitative differences in results by ethnicity.

*LIPA* (lipase A) encodes a lysosomal acid lipase involved in the breakdown of cholesteryl esters and triglycerides. Mutations in *LIPA* cause Wolman's disease [16], a rare disorder characterized by accumulation of these lipids in multiple organs. However, despite evidence that the risk allele was associated with higher *LIPA* gene expression (suggesting that both under- and over-activity of LIPA increase CAD risk), it was not significantly associated with altered lipid levels. This finding suggests that the impact on CAD risk is either through an alternative pathway, or that the mechanism is more complex than reflected through conventionally measured plasma lipid levels. Two recent studies have also found associations of variants in the *LIPA* gene with CAD using a GWA approach, strengthening the evidence for this association [17], [18].

Our identification of the association of variants near interleukin 5 (*IL5*), an interleukin produced by T helper-2 cells, is interesting given the evidence that both acute and chronic inflammation may play important roles in the development and progression of CAD [19]. Most previous human association studies of inflammatory genes and CAD have focused on other cytokines and acute-phase reactants. Nevertheless, some experimental data predict that IL-5 has an atheroprotective effect and this has been supported by association between higher circulating IL-5 levels and lower carotid intimal-medial thickness [20]–[22]. Our findings now highlight the potential importance of IL-5 in CAD, especially as the IL-5 receptor is already a viable therapeutic target in allergic diseases, although we can not rule out the possibility that another gene at this locus may be mediating the association with CAD risk.

The ATP-binding cassette sub-family G proteins ABCG5 and ABCG8 are hemi-transporters that limit intestinal absorption and promote biliary excretion of sterols. Mutations in either gene are associated with sitosterolaemia, accumulation of dietary cholesterol and premature atherosclerosis [23]. Recently, common variants in *ABCG8* have also been shown to be associated with circulating LDL-C and altered serum phytosterol levels with concordant changes in risk of CAD [15], [24]. Our findings confirm that this locus affects CAD risk either directly through its effect on plasma phytosterol levels or through primary/secondary changes in LDL-cholesterol.

The association signal on 8q24.13 maps near the *TRIB1* gene which encodes the Tribbles homolog 1 protein. Tribbles are a family of phosphoproteins implicated in regulation of cell function, although their precise roles are unclear [25]. However, SNPs in or near *TRIB1* - including the lead SNP in our study (rs17321515) - have recently been shown to have highly significant associations with levels of several major lipids [15], providing a possible mechanism for their association with CAD. Our findings confirm the previous suggestion that this variant is also associated with CAD risk [15], [26]. Hepatic over-expression of *TRIB1* in mice has been shown to lower circulating triglycerides; conversely, targeted deletion of the *TRIB1* gene in mice led to higher circulating triglycerides [27]. The location of the CAD-associated variant downstream of *TRIB1* suggests that its effect may be mediated by regulation of *TRIB1* expression leading to adverse lipid profiles, although we did not find an eQTL at this locus in monocytes.

**Figure 7:**
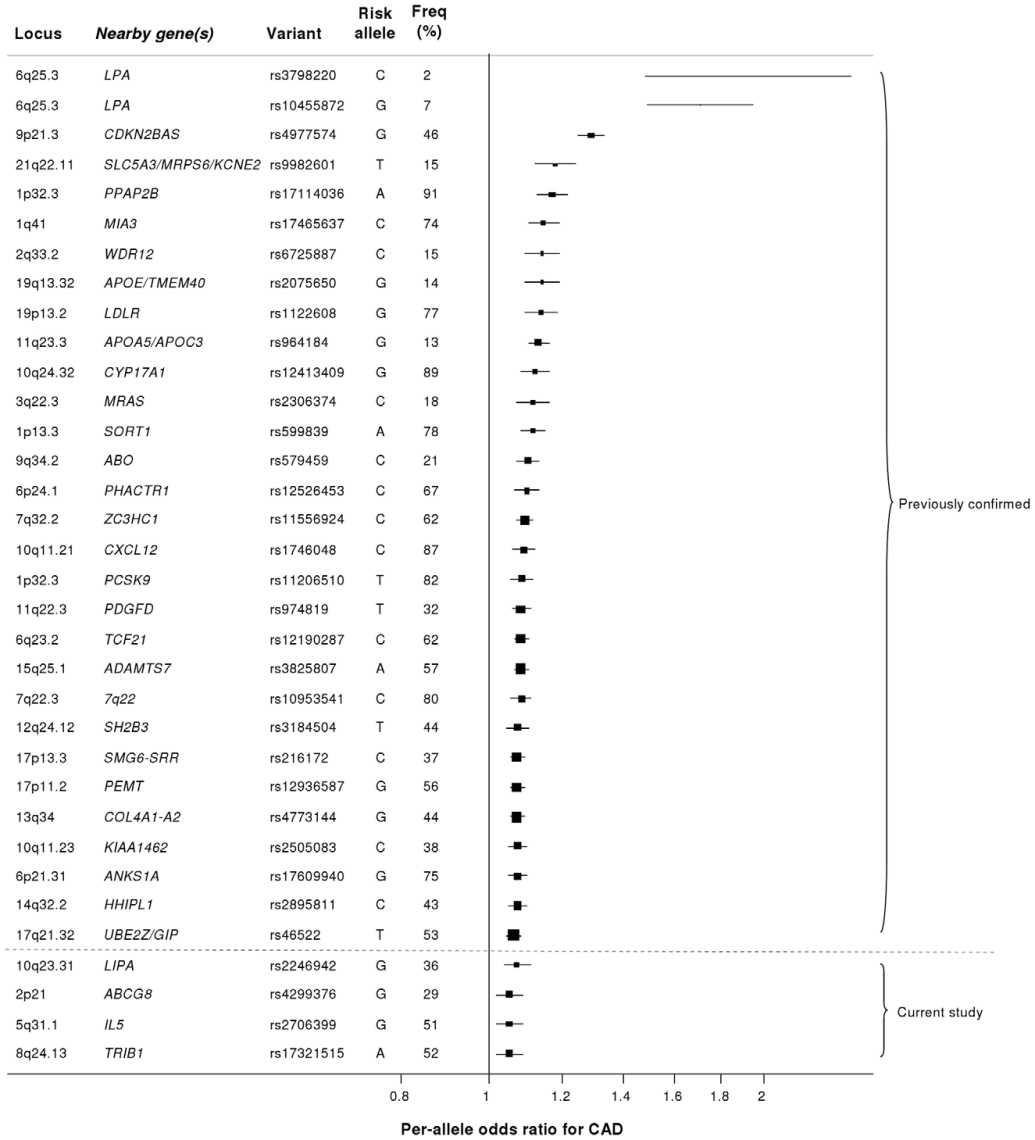
Novel loci identified in this study placed in the context of previously confirmed CAD loci. Previously reported variants listed are those from the NHGRI GWA studies catalogue [32] reported as having P<5×10^−8^ with CAD. Per-allele odds ratios and percentage risk allele frequencies (‘Freq’) are those listed in the catalogue. Frequencies and per-allele odds ratios for the novel variants reported in this study (appearing below the dashed line) are from the CARDIoGRAM replication stage.

Our study brings to 33 the number of confirmed loci with common variants affecting risk of CAD (Figure 7). We estimate that in aggregate these variants explain about 9% of the heritability of CAD which is consistent with the recent analysis by CARDIoGRAM [12]. Interestingly, the odds ratios that we observed for the novel loci were generally lower than those of previously identified loci. This suggests that most of the common variants with moderate effects have been identified and that increasingly larger sample sizes will be required to detect further common variants that affect risk of CAD. However, the modest odds ratios associated with such variants do not necessarily imply that they are not of potential clinical or therapeutic relevance. For example, there are only modest effects of common variants in the *LDLR* gene on CAD risk (Figure 7); yet this pathway has become a major target for the prevention and treatment of CAD with the development of statins.

Despite the success of the GWA approach in identifying several common variants that affect risk of CAD, such loci explain only a small proportion of the heritability of CAD [5]. It has been hypothesized that some of the unexplained heritability resides in lower frequency (1–5%) variants which are not adequately represented on current genomewide arrays and/or are difficult to impute from GWA data. Because the gene array used in the current study included ∼4500 lower frequency variants as well as known functional variants for the majority of the genes on the array, we were able to examine this issue for CAD, at least in relation to candidate cardiovascular genes. Although we confirmed the previously reported associations of lower frequency variants in *LPA* and *PCSK9* with CAD risk, we did not detect any other strongly associated variants in the 1–5% range or an enrichment of low frequency variants amongst SNPs that showed nominal association with CAD. However, it is important to note that rare variants in the genome (minor allele frequency <1%) were not addressed in this study.

CAD is more common in South Asians and tends to occur at an earlier age than in Europeans, perhaps partly due to genetic factors [28]. Our study provides the first systematic exploration of this issue. We observed a weaker effect size for the 9p21.3 locus in South Asians compared with Europeans, although this did not appear to be related to any obvious differences in haplotype structure at the locus, confirming recent findings in Pakistanis [29]. This difference in effect size between ethnic groups will require further evaluation and replication as other differences between the European and South Asian studies (eg, different sex distributions) could explain this finding. Most of the other disease-associated variants we found had slightly weaker effects in South Asians, although, because power to detect heterogeneity of effect between the ethnicities was low and there were only 2 South Asian studies, this finding will require further evaluation. We observed variants at 3 loci (*TUB*, *LCT* and *MICB*, Table S5) which showed modest (P<10^−4^) associations in South Asians but were convincingly null in Europeans and will therefore require replication in additional South Asian samples. Overall, we did not find clear evidence of major variation in genetic risk factors for CAD between Europeans and South Asians.

In summary, using a large-scale gene-centric approach we have identified novel associations of several genes for CAD that relate to diverse biochemical and cellular functions, including inflammation and novel lipid pathways, as well as genes of less certain function. Together, these findings indicate that previously unsuspected biological mechanisms operate in CAD, raising prospects for novel approaches to intervention.

## Materials and Methods

### Participants

Characteristics of the discovery phase studies are summarised in Table 1, Table S1 and the replication studies in Table S6. Further details of all the studies are given in Text S1. All individuals provided informed consent and all studies were approved by local ethics committees.

### Genotyping in discovery cohorts

Using the HumanCVD BeadChip array (Illumina), which is also known as the “ITMAT-Broad-CARe” (IBC) 50K array, we genotyped 49,094 single nucleotide polymorphisms (SNPs) in ∼2,100 candidate genes identified in previous studies of cardiovascular disease, pathway-based approaches (including genes related to metabolism, lipids, thrombosis, circulation and inflammation), early access to GWA datasets for CAD, type 2 diabetes, lipids and hypertension, as well as human and mouse gene expression data [10]. Variants in genes suspected to be associated with sleep, lung and blood disease phenotypes were also included, along with SNPs that were related in GWA datasets to rheumatoid arthritis, Crohn's disease and type 1 diabetes. Human and mouse gene expression data was also used to select variants. Genes were then prioritised by investigators, with ‘high priority genes’ densely tagged (all SNPs with MAF>2% tagged at r^2^>0.8), ‘intermediate priority genes’ moderately covered (all SNPs with MAF>5% tagged at r^2^>0.5), and ‘low priority genes’ tagged using only non-synonymous SNPs and known functional variants with MAF>1%.

A “cosmopolitan tagging” approach was used to select SNPs providing high coverage of selected genes in 4 HapMap populations (CEPH Caucasians, Han Chinese, Japanese, Yorubans). For all genes, non-synonymous SNPs and known functional variants were prioritised on the array. Genotypes were called using standard algorithms (eg, GenCall Software and Illuminus) and standard quality control methods were applied to filter out poorly performing or rare (<1% minor allele frequency) SNPs (Text S1). After exclusion of low frequency variants (average 8,354 in each study), non-autosomal variants (average 1,224) and variants that failed quality control (average 842 – predominantly due to high missingness or failure of HWE), the number of SNPs taken forward for analysis in each study ranged from 30,550–39,027 (Table S2).

### Statistical analysis

In each study, unadjusted logistic regression tests using a case-control design assuming an additive genetic model were conducted, with most studies using PLINK [30]. All studies made attempts to reduce over-dispersion. The genomic inflation factor for each study after adjustment was <1.10 with one exception (Table S2). The primary analysis was a fixed-effect inverse-variance-weighted meta-analysis performed separately for each ethnic group using STATA v11. A chi-squared test for between-ethnicity heterogeneity was performed. A secondary analysis combined European and South Asian studies to identify additional variants common to both ethnicities (Text S1).

### Replication

Based on a simulation study conducted prior to the analysis (Figure S2), variants were selected for the replication stage if they had an unadjusted P<1×10^−4^ in either the primary analysis or the combined ethnicity analysis. Only the most significant (“lead”) SNP from each locus was taken forward for replication. SNPs at known coronary disease risk loci (eg, 9p21.3, *LPA*, *APOE*) were excluded from the replication stage, leaving 27 SNPs to take forward. *In silico* replication was conducted using *non-overlapping* participants from the CARDIoGRAM GWA meta-analysis [12] of CAD plus an additional study, EPIC-NL [31] (details in Table S6). In total, the replication stage comprised up to 17,121 coronary disease cases and 40,473 controls. The threshold for independent replication was a 1-tailed Bonferroni-corrected P<0.05 (P<1.9×10^−3^) from a Cochran-Armitage trend test. P values from the replication and discovery stages were combined using Fisher's method with a chip-wide value of P<3×10^−6^ considered to be statistically significant based on the simulation study (Figure S2). Adjusted P values accounting for both over-dispersion and heterogeneity in the discovery stage studies were also estimated through correction for study- and meta-analysis-specific inflation factors.

### Additional analyses

To check for consistency of effect of variants that replicated, subgroup analyses were performed in the discovery stage studies for MI cases only, CAD cases aged less than 50, males only and females only. Replicating SNPs were tested for association with known cardiovascular risk factors such as blood pressure, lipids levels and type 2 diabetes mellitus using existing large-scale GWA meta-analyses data of these traits [13]–[15]. We also assessed the association of these variants with gene expression in circulating monocytes taken from 363 patients with premature myocardial infarction and 395 healthy blood donors (Text S1). To put novel findings from this study in the context of existing knowledge, we summarised associations of common variants established in CAD (P<5×10^−8^) using available information from the NHGRI's GWA studies catalogue [32].

## Supporting Information

Figure S1 Power to detect associated variants in discovery and replication stages. Power to detect an association with alpha = 10^−4^ (two-sided) assuming a per-allele effect and a discovery stage study size of 11,202 coronary disease cases and 30,733 controls (equivalent to the European studies in the discovery stage) across a range of minor allele frequencies (1%, 2%, 3%, 4%, 5%, 10%). These power calculations assume that there is no between-study heterogeneity. Power to detect an association with alpha = 1.9×10^−3^ (one-sided) assuming a per-allele effect and a replication stage study size of 17,121 coronary disease cases and 40,473 controls (equivalent to the whole replication stage) range of minor allele frequencies (5%, 10%, 25%, 50%). These power calculations assume that there is no between-study heterogeneity.
(PDF)

Figure S2 Simulated distribution of P values from discovery stage meta-analyses. The distribution of the number of SNPs with a P value<10^−4^ under the null hypothesis of no associated SNPs is based on 50,000 simulations using the controls from the BHF-FHS study. The median is 2 significant SNPs (mean 2.5), suggesting that using this threshold for taking SNPs to the replication stage is likely to result in few false positives. The comparable numbers for a threshold of P<10^−3^ are median = 27 (mean 27), whilst the mean was 0.25 for P<10^−5^. The distribution of lowest P value in each simulation across the Human CVD Beadchip array is based on 50,000 simulations using the controls from the BHF-FHS study. The vertical line at P = 3×10^−6^ represents the 5th percentile, which was selected to denote chip-wide significance.
(PDF)

Figure S3 Forest plots for novel SNPs in discovery stage studies. Forest plots denote study-specific per-allele estimates of risk of CAD, with the centre of each box representing the odds ratio, the area of the box proportional to the weight (the inverse of the variance), and the horizontal line indicating the 95% confidence interval. Log odds ratios and standard errors were pooled using a fixed-effect meta-analysis. Open diamonds represent pooled estimates and 95% confidence intervals. European and South Asian subgroup analyses did not differ significantly from each other for any of the SNPs displayed.
(PDF)

Figure S4 Subgroup analyses for novel loci in European discovery stage studies. Allele = Allele associated with increased risk of CAD; Freq = frequency of risk allele in control populations. MI = MI cases only vs all controls; Young = CAD cases diagnosed aged less than 50 years.
(PDF)

Table S1 Details of studies included in the discovery stage. - denotes ‘not applicable’ or ‘not available’. All values are means (±SD) unless otherwise stated. Percentages may not be of all available individuals due to missing data. ARIC = Atherosclerosis Risk In Communities; BHF-FHS = British Heart Foundation Family Heart Study; CARDIA = Coronary Artery Risk Development in Young Adults; CHS = Cardiovascular Health Study; FHS = Framingham Heart Study; LOLIPOP = London Life Sciences Prospective Population Cohort; PROCARDIS = Precocious Coronary Artery Disease; PROMIS = Pakistan Risk of Myocardial Infarction Study. * age at baseline. 4 studies (BHF-FHS, MONICA-KORA, PennCATH and PROMIS) used version 1 (V1) of the array, whilst the other 8 used version 2 (V2). V2 contains an additional 132 genes (3,857 SNPs) hence SNPs on V2 were only analysed in studies that used the V2 array. Participants in the Framingham Heart Study were drawn from the Offspring and Third Generation cohorts.
(PDF)

Table S2 Quality control information for SNPs in discovery stage studies. Inflation factor = ratio of median observed chi^2^ value to that expected under the null hypothesis; MAF = minor allele frequency; No result = no odds ratio obtained from model, generally due to low MAF; HWE = Hardy-Weinberg equilibrium (P value estimated for controls only).
(PDF)

Table S3 Results for all loci meeting P<10^−3^ in discovery stage meta-analyses. SNPs are ordered by ascending P value in the combined meta-analysis. Only the lead SNP (with the lowest P value) from each locus is shown unless different SNPs met the threshold in Europeans/South Asians. Data shown are per-allele odds ratios from unadjusted fixed-effect inverse-variance meta-analysis of 10 European studies, 2 South Asians studies and 12 studies combined. Loci highlighted in grey are those previously identified by GWA studies; loci highlighted in yellow are additional loci considered to be known CAD risk loci.
(PDF)

Table S4 Associations with previously studied candidate variants. Variants ordered by biological pathway, then gene. Per-allele odds ratios are presented for the effect allele, which is the minor allele in European populations. r^2^ with best imputed proxy was estimated using ∼2.5 M directly genotyped or HapMap-imputed SNPs in the CARDIoGRAM Consortium. Tagging levels are 1 (r^2^>0.8 with all HapMap/Seattle SNPs of MAF≥0.02), 2 (r^2^>0.5 with all HapMap/Seattle SNPs of MAF≥0.05), 3 (only non-synonymous and known functional variants of MAF>0.01) and GWAS (specific SNPs previously identified in recent GWAS). ^a^ rs4343 has r^2^ = 1 with the insertion/deletion polymorphism in the *ACE* gene in CEU HapMap 2 population. ^b^ rs17443251 has r^2^ = 0.75 with the more commonly studied R144C variant (rs1799853) in the *CYP2C9* gene in CEU HapMap 2 population. ^c^ rs9526246 has r^2^ = 0.97 with the more commonly studied T102C variant (rs6313) in the *HTR2A* gene in CEU HapMap 2 population. ^d^ rs1062535 has r^2^ = 0.97 with the more commonly studied C807T variant (rs1126643) in the *ITGA2* gene in CEU HapMap 2 population. ^e^ rs1805096 has r^2^ = 0.89 with the more commonly studied rs6700896 variant in the *LEPR* gene in CEU HapMap 2 population. ^f^ rs1049897 has r^2^ = 1 with the more commonly studied A102T variant (rs4236) in the *MGP* gene in CEU HapMap 2 population. ^g^ rs4968624 has r^2^ = 0.97 with the more commonly studied L125V variant (rs668) in the *PECAM1* gene in CEU HapMap 2 population. ^h^ rs12944077 has r^2^ = 1 with the more commonly studied S563N variant (rs12953) in the *PECAM1* gene in CEU HapMap 2 population.
(PDF)

Table S5 27 loci meeting P<1×10^−4^ threshold in discovery stage meta-analyses. Lead SNP = SNP with lowest P-value at this locus; risk allele = allele associated with increased CAD risk according to forward strand; freq = frequency of risk allele pooled across controls; OR = per-allele odds ratio for risk allele; 95% CI = 95% confidence interval around odds ratio; P = P value from fixed-effect meta-analysis; Combined = 10 European studies and 2 South Asian studies combined in a single fixed-effect meta-analysis; Overall = P values from discovery stage and replication stage combined; P_adj = P value adjusted for both study-specific and meta-analysis inflation factors in the discovery stage; SNPs ordered by ascending P value. * For 3 loci (*ZC3HC1*, *CYP17A1*, *COL4A1/COL4A2*), replication data are not presented here, however genome-wide significant associations at these loci are reported in the paper by the CARDIoGRAM Consortium.
(PDF)

Table S6 Details of studies included in the replication stage. All values are means (±SD) unless otherwise stated.
(PDF)

Table S7 Expression QTL (eQTL) analysis for novel CAD loci. a. eQTL analysis for novel CAD loci. †Key (Proportion of all Probes). 1 = Weak (0%–20%). 2 = Medium (20%–80%). 3 = Strong (80%–100%). b. Conditional analysis of expression QTL (eQTL) loci. Conditional analysis of the lead *LIPA* SNP on a secondary SNP at the same locus that is also associated with gene expression shows that the lead SNP at the *LIPA* locus has a strong independent effect on *LIPA* expression levels. Conditional analysis of the lead *IL5* SNP on a second nearby SNP that is also associated with *RAD50* gene expression shows that the observed eQTL association with the *IL5* SNP is probably due to LD with the *RAD50* SNP.
(PDF)

Table S8 Comparison of haplotype frequencies for novel loci in European and South Asian controls. Haplotypes are displayed in decreasing frequency, with the same haplotype order in both ethnicities. * = haplotype frequencies in bold are those containing the CAD risk-associated allele of the lead SNP. SNPs were selected for inclusion in the haplotype if they had r^2^≥0.5 in either the European or the South Asian controls. The 3330 PROCARDIS controls were used to represent the European populations, whilst the PROMIS controls were used to represent the South Asian population. Only haplotypes that were common (frequency>5%) in at least one population are displayed.
(PDF)

Text S1 Supplementary Methods, Supplementary References, Supplementary Acknowledgements.
(PDF)
