# Correction: Large-Scale Gene-Centric Analysis Identifies Novel Variants for Coronary Artery Disease

**DOI:** 10.1371/annotation/120649cf-8c28-43c9-a688-c7cd65eb1aec

**Published:** 2012-08-14

**Authors:** The IBC 50K CAD Consortium

In the CARDIoGRAM Study Collaborating Group, the 107th author's name was misspelt. The correct spelling is Frits R Rosendaal.

