# Supplementary material for: Large-Scale Gene-Centric Analysis Identifies Novel Variants for Coronary Artery Disease
Source: PLoS Genet. 2011 Sep 22;7(9):e1002260. doi: 10.1371/journal.pgen.1002260 (PMC3178591; doi:10.1371/journal.pgen.1002260)
Supplement: Figure S1 — Power to detect associated variants in discovery and replication stages. Power to detect an association with alpha = 10−4 (two-sided) assuming a per-allele effect and a discovery stage study size of 11,202 coronary disease cases and 30,733 controls (equivalent to the European studies in the discovery stage) across a range of minor allele frequencies (1%, 2%, 3%, 4%, 5%, 10%). These power calculations assume that there is no between-study heterogeneity. Power to detect an association with alpha = 1.9×10^−3^ (one-sided) assuming a per-allele effect and a replication stage study size of 17,121 coronary disease cases and 40,473 controls (equivalent to the whole replication stage) range of minor allele frequencies (5%, 10%, 25%, 50%). These power calculations assume that there is no between-study heterogeneity. (PDF) [file pgen.1002260.s001.pdf]

Figure S1. Power to detect associated variants in discovery and replication stages.

a) Discovery stage meta-analysis.

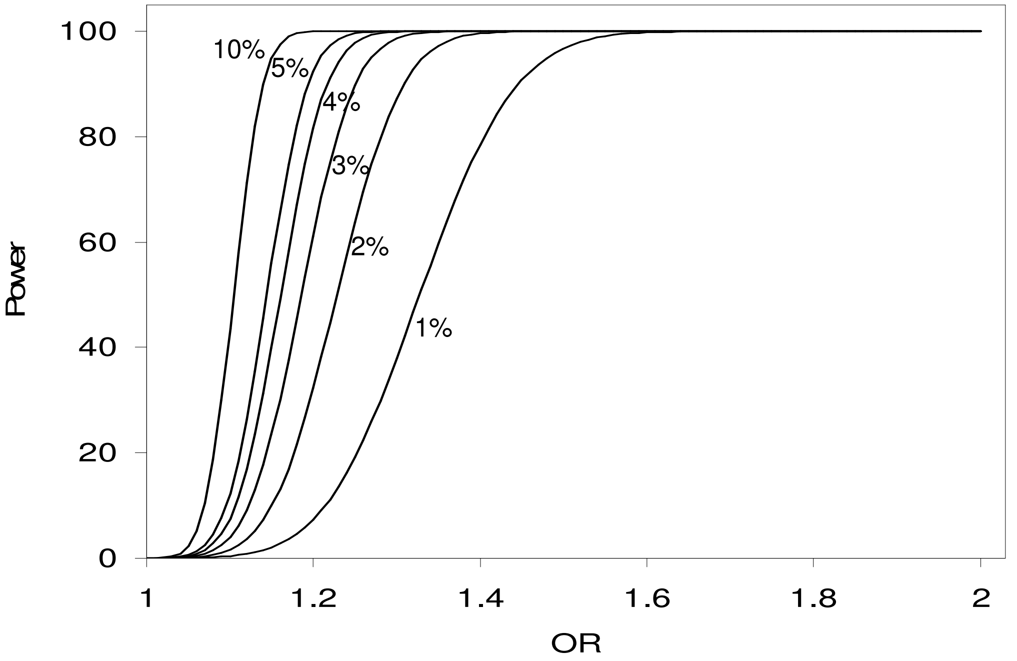

Power to detect an association with alpha = 10^{-4} (two-sided) assuming a per-allele effect and a discovery stage study size of 11,202 coronary disease cases and 30,733 controls (equivalent to the European studies in the discovery stage) across a range of minor allele frequencies (1%,2%,3%,4%,5%,10%). These power calculations assume that there is no between-study heterogeneity.

b) Replication stage meta-analysis.

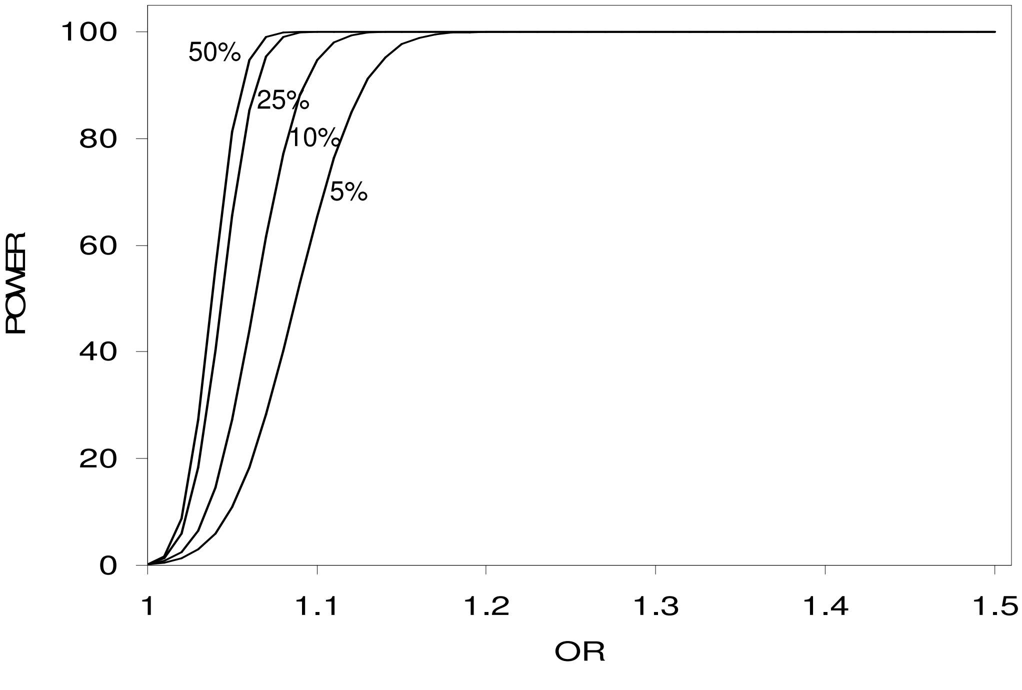

Power to detect an association with alpha = 1.9×10^{-3} (one-sided) assuming a per-allele effect and a replication stage study size of 17,121 coronary disease cases and 40,473 controls (equivalent to the whole replication stage) range of minor allele frequencies (5%,10%,25%,50%). These power calculations assume that there is no between-study heterogeneity.
