# Supplementary material for: Large-Scale Gene-Centric Analysis Identifies Novel Variants for Coronary Artery Disease
Source: PLoS Genet. 2011 Sep 22;7(9):e1002260. doi: 10.1371/journal.pgen.1002260 (PMC3178591; doi:10.1371/journal.pgen.1002260)
Supplement: Figure S2 — Simulated distribution of P values from discovery stage meta-analyses. The distribution of the number of SNPs with a P value<10−4 under the null hypothesis of no associated SNPs is based on 50,000 simulations using the controls from the BHF-FHS study. The median is 2 significant SNPs (mean 2.5), suggesting that using this threshold for taking SNPs to the replication stage is likely to result in few false positives. The comparable numbers for a threshold of P<10−3 are median = 27 (mean 27), whilst the mean was 0.25 for P<10−5. The distribution of lowest P value in each simulation across the Human CVD Beadchip array is based on 50,000 simulations using the controls from the BHF-FHS study. The vertical line at P = 3×10^−6^ represents the 5th percentile, which was selected to denote chip-wide significance. (PDF) [file pgen.1002260.s002.pdf]

Figure S2. Simulated distribution of P values from discovery stage meta-analyses.

a) Determination of replication threshold

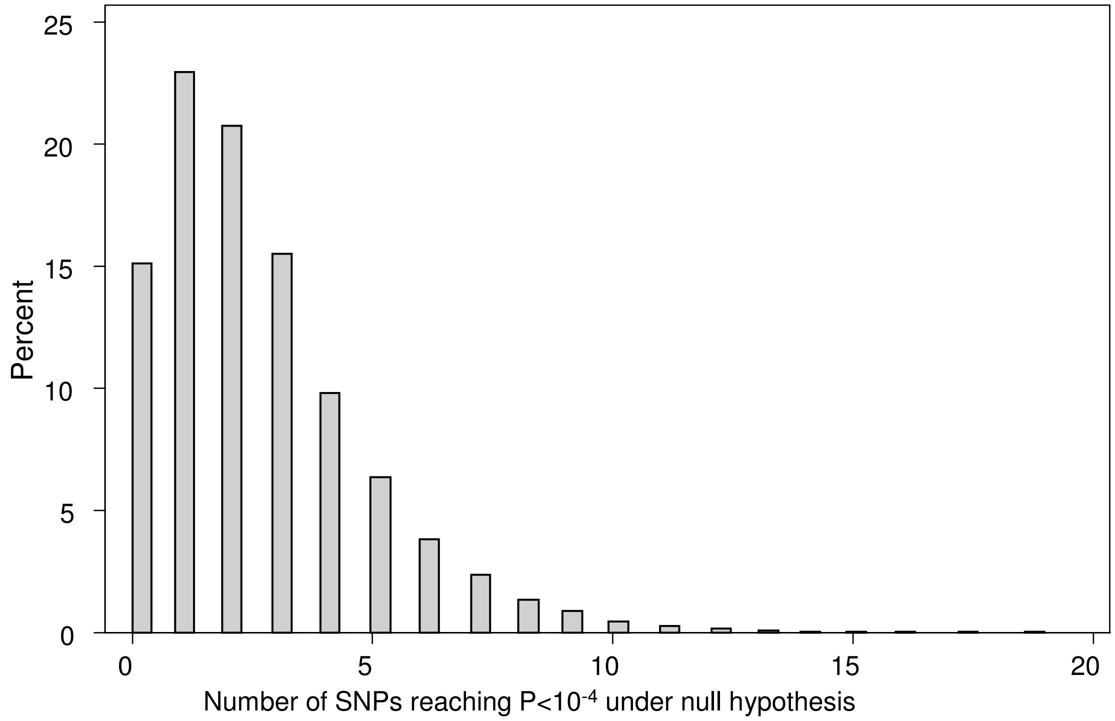

The distribution of the number of SNPs with a P value$<10^{-4}$ under the null hypothesis of no associated SNPs is based on 50,000 simulations using the controls from the BHF-FHS study. The median is 2 significant SNPs (mean 2.5), suggesting that using this threshold for taking SNPs to the replication stage is likely to result in few false positives. The comparable numbers for a threshold of $P<10^{-3}$ are median=27 (mean 27), whilst the mean was 0.25 for $P<10^{-5}$.

b) Chip-wide significance threshold

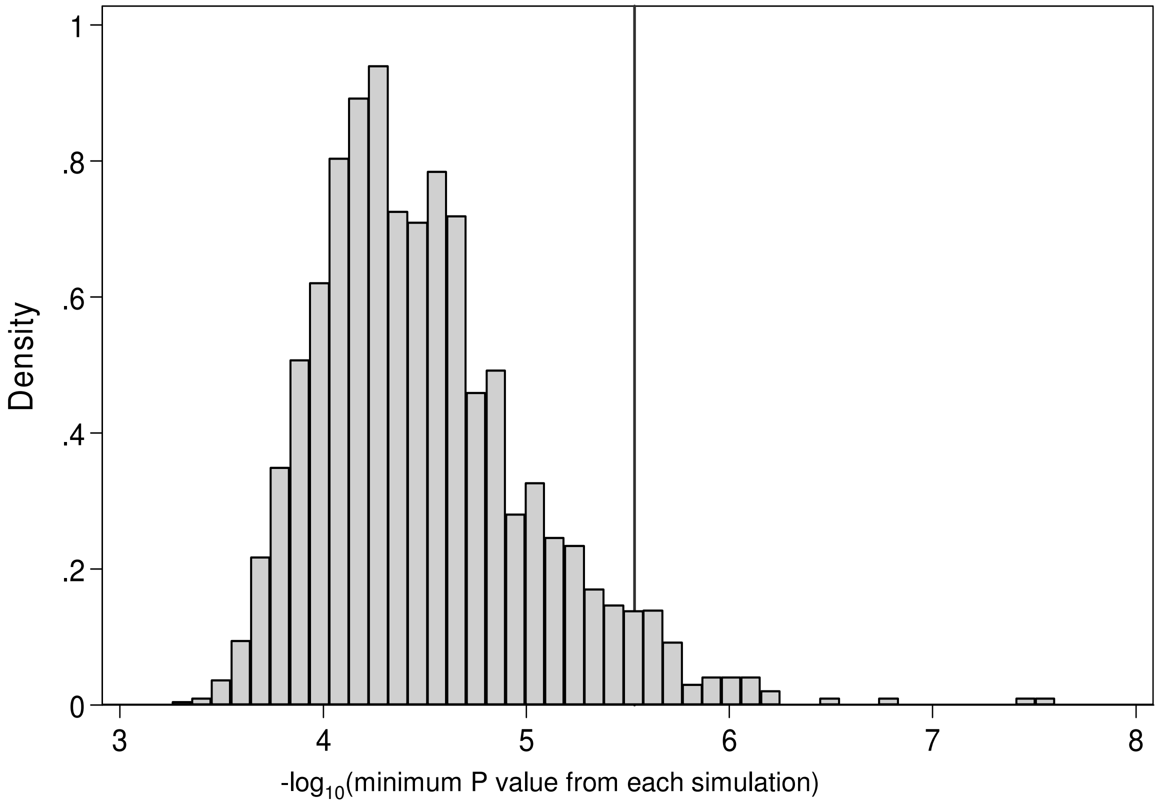

The distribution of lowest P value in each simulation across the Human CVD Beadchip array is based on 50,000 simulations using the controls from the BHF-FHS study. The vertical line at $P=3x10^{-6}$ represents the 5th percentile, which was selected to denote chip-wide significance.
