# Supplementary material for: Large-Scale Gene-Centric Analysis Identifies Novel Variants for Coronary Artery Disease
Source: PLoS Genet. 2011 Sep 22;7(9):e1002260. doi: 10.1371/journal.pgen.1002260 (PMC3178591; doi:10.1371/journal.pgen.1002260)
Supplement: Figure S3 — Forest plots for novel SNPs in discovery stage studies. Forest plots denote study-specific per-allele estimates of risk of CAD, with the centre of each box representing the odds ratio, the area of the box proportional to the weight (the inverse of the variance), and the horizontal line indicating the 95% confidence interval. Log odds ratios and standard errors were pooled using a fixed-effect meta-analysis. Open diamonds represent pooled estimates and 95% confidence intervals. European and South Asian subgroup analyses did not differ significantly from each other for any of the SNPs displayed. (PDF) [file pgen.1002260.s003.pdf]

Figure S3. Forest plots for novel SNPs in discovery stage studies.

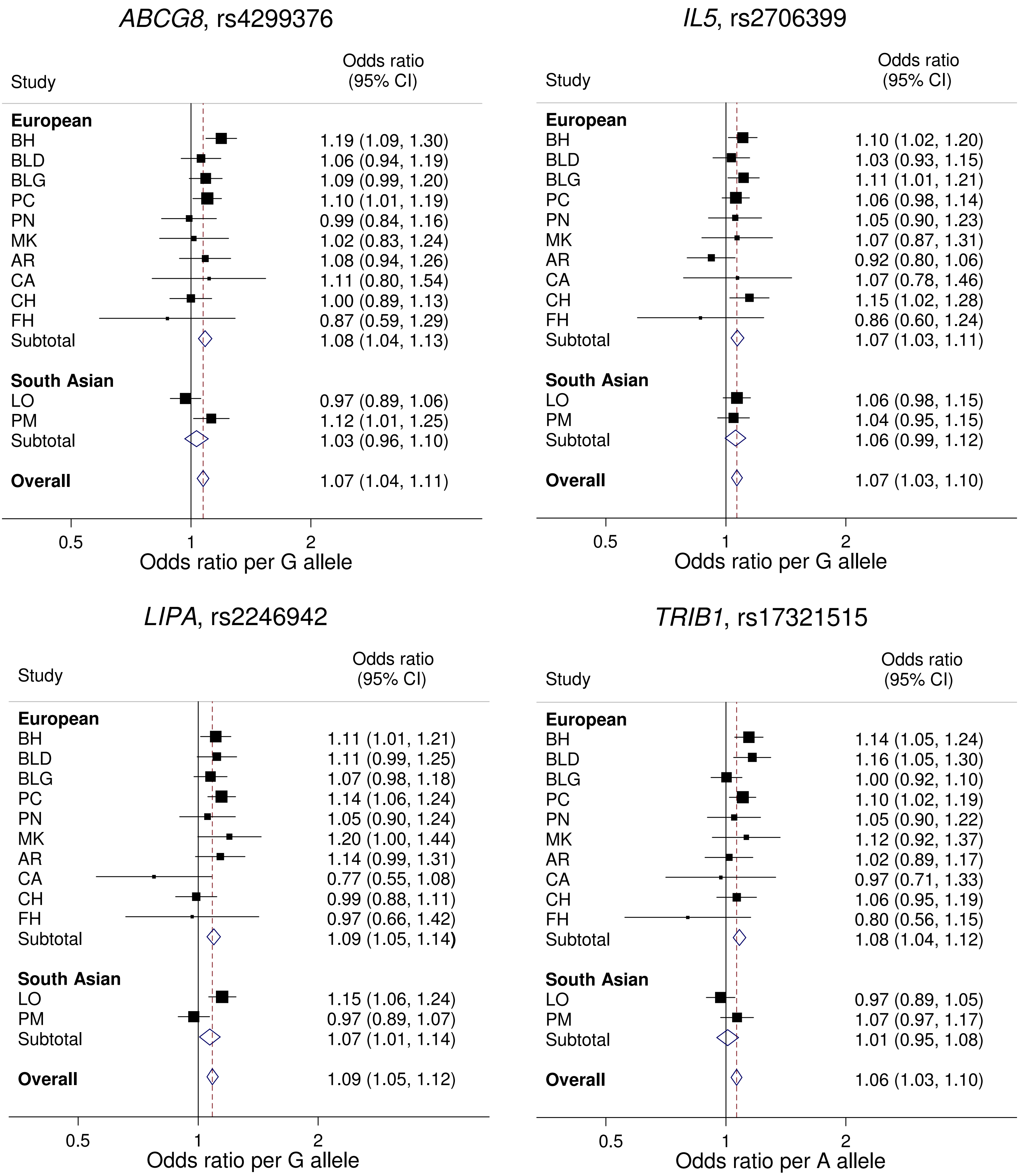

Forest plots denote study-specific per-allele estimates of risk of CAD, with the centre of each box representing the odds ratio, the area of the box proportional to the weight (the inverse of the variance), and the horizontal line indicating the 95% confidence interval. Log odds ratios and standard errors were pooled using a fixed-effect meta-analysis. Open diamonds represent pooled estimates and 95% confidence intervals. European and South Asian subgroup analyses did not differ significantly from each other for any of the SNPs displayed.
