# Supplementary material for: Large-Scale Gene-Centric Analysis Identifies Novel Variants for Coronary Artery Disease
Source: PLoS Genet. 2011 Sep 22;7(9):e1002260. doi: 10.1371/journal.pgen.1002260 (PMC3178591; doi:10.1371/journal.pgen.1002260)
Supplement: Figure S4 — Subgroup analyses for novel loci in European discovery stage studies. Allele = Allele associated with increased risk of CAD; Freq = frequency of risk allele in control populations. MI = MI cases only vs all controls; Young = CAD cases diagnosed aged less than 50 years. (PDF) [file pgen.1002260.s004.pdf]

Figure S4. Subgroup analyses for novel loci in European discovery stage studies.

Gene	SNP	Allele	Freq	Subgroup	Studies	Cases	Controls	Odds ratio (95% CI)
ABCG8	rs4299376	G	0.33	MI	6	7069	10805	1.09 (1.04, 1.15)
				Young	5	2874	8758	1.12 (1.05, 1.20)
				Male	10	8244	14448	1.07 (1.02, 1.13)
				Female	10	2945	16278	1.10 (1.02, 1.17)
				Total	10	11189	30726	1.08 (1.04, 1.13)
IL5	rs2706399	G	0.52	MI	6	7077	10812	1.08 (1.04, 1.13)
				Young	5	2877	8765	1.05 (0.98, 1.12)
				Male	10	8253	14453	1.04 (0.99, 1.09)
				Female	10	2948	16280	1.10 (1.03, 1.17)
				Total	10	11201	30733	1.07 (1.03, 1.11)
TRIB1	rs17321515	A	0.54	MI	6	7078	10810	1.10 (1.05, 1.15)
				Young	5	2878	8763	1.15 (1.08, 1.22)
				Male	10	8253	14452	1.08 (1.03, 1.13)
				Female	10	2949	16278	1.09 (1.02, 1.16)
				Total	10	11202	30730	1.08 (1.04, 1.12)
LIPA	rs2246942	G	0.35	MI	7	7157	12134	1.11 (1.06, 1.16)
				Young	5	2870	8751	1.21 (1.13, 1.29)
				Male	9	8022	13670	1.11 (1.05, 1.16)
				Female	9	2891	15583	1.08 (1.01, 1.15)
				Total	10	11188	30664	1.09 (1.05, 1.14)

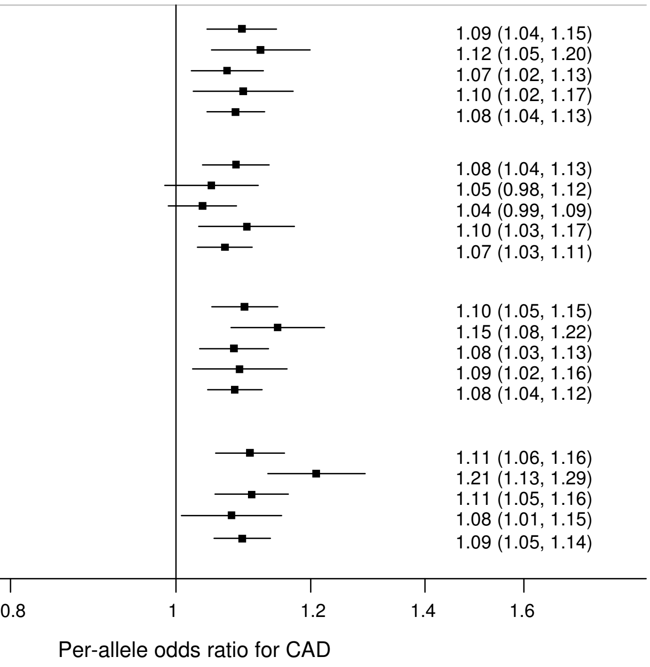

Allele = Allele associated with increased risk of CAD; Freq = frequency of risk allele in control populations.

MI = MI cases only vs all controls; Young = CAD cases diagnosed aged less than 50 years.
