# Supplementary material for: Large-Scale Gene-Centric Analysis Identifies Novel Variants for Coronary Artery Disease
Source: PLoS Genet. 2011 Sep 22;7(9):e1002260. doi: 10.1371/journal.pgen.1002260 (PMC3178591; doi:10.1371/journal.pgen.1002260)
Supplement: Table S1 — Details of studies included in the discovery stage. - denotes ‘not applicable’ or ‘not available’. All values are means (±SD) unless otherwise stated. Percentages may not be of all available individuals due to missing data. ARIC = Atherosclerosis Risk In Communities; BHF-FHS = British Heart Foundation Family Heart Study; CARDIA = Coronary Artery Risk Development in Young Adults; CHS = Cardiovascular Health Study; FHS = Framingham Heart Study; LOLIPOP = London Life Sciences Prospective Population Cohort; PROCARDIS = Precocious Coronary Artery Disease; PROMIS = Pakistan Risk of Myocardial Infarction Study. * age at baseline. 4 studies (BHF-FHS, MONICA-KORA, PennCATH and PROMIS) used version 1 (V1) of the array, whilst the other 8 used version 2 (V2). V2 contains an additional 132 genes (3,857 SNPs) hence SNPs on V2 were only analysed in studies that used the V2 array. Participants in the Framingham Heart Study were drawn from the Offspring and Third Generation cohorts. (PDF) [file pgen.1002260.s005.pdf]

Table S1. Details of studies included in the discovery stage.

	European discovery													
	ARIC		BHF-FHS		BLOODOMICS Dutch		BLOODOMICS German		CARDIA		CHS		FHS	
Status	Cases	Controls	Cases	Controls	Cases	Controls	Cases	Controls	Cases	Controls	Cases	Controls	Cases	Controls
n	448	8873	2101	2426	1462	1222	1910	1932	86	1346	755	3197	59	6976
Age (yrs)	57.2 (5.3)	54.1 (5.7)	60.1 (8.1)	42.2 (12.4)	-	-	-	-	25.9 (3.2)	25.6 (3.4)	73.9 (5.7)	72.5 (5.5)	54.5 (8.6)	43.2 (10.9)
Age at diagnosis (yrs)	-	-	49.8 (7.7)	-	48.8 (12.0)	-	59.3 (10.9)	-	-	-	-	-	-	-
Male (n, %)	363 (81.0)	3974 (44.8)	1655 (78.8)	1211 (49.9)	1066 (77.4)	825 (67.5)	1456 (76.2)	1932 (50.5)	30 (34.9)	640 (47.6)	449 (59.5)	1283 (40.1)	41 (69.5)	3133 (44.9)
MI (n, %)	368 (82.7)	-	1538 (73.2)	-	1462 (100)	-	1181 (61.8)	-	86 (100)	-	381 (50.5)	-	13 (22.0)	-
Type 2 diabetes (n, %)	90 (20.1)	719 (8.1)	235 (11.2)	-	68 (6.6)	-	-	-	3 (3.5)	7 (0.6)	165 (21.9)	411 (12.9)	3 (5.1)	160 (2.3)
Hypertension (n, %)	211 (47.7)	2275 (25.7)	920 (43.8)	-	378 (29.2)	-	-	-	10 (11.6)	103 (7.7)	391 (51.9)	1264 (39.6)	31 (53.5)	1378 (19.8)
Family history (n, %)	252 (56.3)	3816 (43.0)	2101 (100)	-	774 (59.5)	-	-	-	12 (14.0)	219 (16.3)	313 (44.5)	909 (31.1)	-	-
BMI (kg/m^2)	28.0 (4.6)	26.9 (4.8)	27.7 (4.3)	-	26.7 (3.8)	-	27.4 (4.0)	-	22.7 (3.5)	23.7 (4.0)	26.5 (4.3)	26.3 (4.5)	26.9 (4.4)	26.2 (5.0)
Current smokers (n, %)	114 (25.4)	2140 (24.1)	914 (43.5)	-	880 (72.5)	-	1347 (70.5)	-	-	321 (24.0)	-	375 (11.7)	18 (30.5)	1580 (22.7)

	European discovery					
	MONICA-KORA		PennCATH		PROCARDIS	
Status	Cases	Controls	Cases	Controls	Cases	Controls
n	275	1413	1027	489	3120	3330
Age (yrs)	57.5 (8.1)	52.1 (10.5)	58.1 (9.8)	60.7 (10.3)	62.7 (7.1)	59.4 (9.9)
Age at diagnosis (yrs)	64.1 (7.6)	-	54.2 (8.8)	-	53.6 (7.6)	-
Male (n, %)	218 (79.3)	753 (53.3)	754 (73.4)	246 (50.3)	2190 (70.2)	1628 (48.9)
MI (n, %)	>50%	-	435 (42.4)	-	2136 (68.5)	-
Type 2 diabetes (n, %)	48 (17.5)	64 (4.5)	218 (21.2)	56 (11.5)	501 (16.1)	114 (3.4)
Hypertension (n, %)	177 (64.4)	580 (41.1)	620 (60.4)	231 (47.2)	1592 (51.0)	821 (24.7)
Family history (n, %)	62 (22.6)	275 (19.5)	475 (46.3)	130 (26.6)	3120 (100)	0 (0)
BMI (kg/m^2)	28.6 (4.0)	27.1 (4.0)	29.5 (5.5)	28.9 (6.3)	28.6 (4.6)	26.8 (4.4)
Current smokers (n, %)	112 (40.7)	350 (24.8)	449 (43.7)	170 (34.8)	2263 (72.5)	1542 (46.3)

	South Asian discovery			
	PROMIS		LOLIPOP	
Status	Cases	Controls	Cases	Controls
n	1856	1905	2538	2354
Age (yrs)	54.5 (10.9)	52.1 (10.3)	58.4 (9.4)	56.6 (8.1)
Age at diagnosis (yrs)	54.5 (10.9)	-	-	-
Male (n, %)	1558 (83.9)	1546 (81.2)	2127 (90.3)	1990 (78.4)
MI (n, %)	1856 (100)	-	1125 (44.4)	-
Type 2 diabetes (n, %)	362 (20.4)	278 (14.7)	994 (39.2)	482 (20.5)
Hypertension (n, %)	1114 (60.0)	801 (42.0)	1637 (64.5)	716 (30.5)
Family history (n, %)	371 (20.9)	203 (10.7)	912 (36.0)	444 (18.9)
BMI (kg/m^2)	25.2 (4.4)	25.3 (4.3)	27.6 (4.4)	26.8 (4.1)
Current smokers (n, %)	934 (53.0)	753 (40.1)	229 (9.0)	214 (9.1)

- denotes 'not applicable' or 'not available'

All values are means (±SD) unless otherwise stated. Percentages may not be of all available individuals due to missing data.

ARIC = Atherosclerosis Risk In Communities; BHF-FHS = British Heart Foundation Family Heart Study; CARDIA = Coronary Artery Risk Development in Young Adults; CHS = Cardiovascular Health Study; FHS = Framingham Heart Study; LOLIPOP = London Life Sciences Prospective Population Cohort; PROCARDIS = Precocious Coronary Artery Disease; PROMIS = Pakistan Risk of Myocardial Infarction Study.

* age at baseline

4 studies (BHF-FHS, MONICA-KORA, PennCATH and PROMIS) used version 1 (V1) of the array, whilst the other 8 used version 2 (V2). V2 contains an additional 132 genes (3,857 SNPs) hence SNPs on V2 were only analysed in studies that used the V2 array.

Participants in the Framingham Heart Study were drawn from the Offspring and Third Generation cohorts.
