# Supplementary material for: Large-Scale Gene-Centric Analysis Identifies Novel Variants for Coronary Artery Disease
Source: PLoS Genet. 2011 Sep 22;7(9):e1002260. doi: 10.1371/journal.pgen.1002260 (PMC3178591; doi:10.1371/journal.pgen.1002260)
Supplement: Table S2 — Quality control information for SNPs in discovery stage studies. Inflation factor = ratio of median observed chi2 value to that expected under the null hypothesis; MAF = minor allele frequency; No result = no odds ratio obtained from model, generally due to low MAF; HWE = Hardy-Weinberg equilibrium (P value estimated for controls only). (PDF) [file pgen.1002260.s006.pdf]

Table S2. Quality control information for SNPs in discovery stage studies.

Studies	ARIC	BHF-FHS	BLOODOMICS Dutch	BLOODOMICS German	CARDIA	CHS	FHS	LOLIPOP	MONICA-KORA	PennCATH	PROCARDIS	PROMIS
**Missingness>2%**	488	1,005	1,048	890	1,215	670	691	953	2,816	2,938	1,181	794
**MAF = 0**	2,246	4,449	6,078	5,461	4,719	3,405	5,773	5,462	6,831	4,487	4,096	3,882
**No result**	5,433	2,884	2,918	2,670	5,304	3,585	5,387	2,602	4,421	3,190	2,518	2,442
**Non-autosomal**	778	801	768	800	707	796	696	818	704	0	741	748
**HWE P<0.0001**	22	99	290	284	16	22	10	232	229	32	112	402
**Inflation factor**	1.031	1.081	1.097	1.041	1.003	1.038	1.022	1.145	1.017	1.020	1.062	1.028
**SNPs entering analysis**	**36,724**	**36,469**	**38,012**	**38,950**	**34,545**	**37,945**	**34,480**	**39,027**	**30,706**	**35,023**	**39,285**	**37,387**

Inflation factor = ratio of median observed chi^2 value to that expected under the null hypothesis; MAF = minor allele frequency; No result = no odds ratio obtained from model, generally due to low MAF;

HWE = Hardy-Weinberg equilibrium (P value estimated for controls only).
