# Supplementary material for: Large-Scale Gene-Centric Analysis Identifies Novel Variants for Coronary Artery Disease
Source: PLoS Genet. 2011 Sep 22;7(9):e1002260. doi: 10.1371/journal.pgen.1002260 (PMC3178591; doi:10.1371/journal.pgen.1002260)
Supplement: Table S3 — Results for all loci meeting P<10−3 in discovery stage meta-analyses. SNPs are ordered by ascending P value in the combined meta-analysis. Only the lead SNP (with the lowest P value) from each locus is shown unless different SNPs met the threshold in Europeans/South Asians. Data shown are per-allele odds ratios from unadjusted fixed-effect inverse-variance meta-analysis of 10 European studies, 2 South Asians studies and 12 studies combined. Loci highlighted in grey are those previously identified by GWA studies; loci highlighted in yellow are additional loci considered to be known CAD risk loci. (PDF) [file pgen.1002260.s007.pdf]

Table S3. Results for all loci meeting P<10^{-3} in discovery stage meta-analyses.

					European studies					South Asian studies					Combined studies			
chr	*Nearest gene(s)*	SNP	Position	Allele	Freq	OR	95% CI		P	Freq	OR	95% CI		P	OR	95% CI		P
9	*CDKN2A/CDKN2B*	rs1333042	22,093,813	A	0.48	0.79	0.76	0.82	5.4E-36	0.41	0.88	0.83	0.94	4.3E-05	0.81	0.79	0.84	1.1E-37
9	*CDKN2A/CDKN2B*	rs944797	22,105,286	T	0.47	0.80	0.77	0.83	4.5E-32	0.46	0.87	0.82	0.93	1.2E-05	0.82	0.79	0.85	5.2E-35
6	*LPA/SLC22A2*	rs10455872	160,930,108	G	0.07	1.45	1.34	1.57	1.5E-20	0.01	1.23	0.84	1.78	2.8E-01	1.44	1.33	1.55	1.2E-20
1	*CELSR2/PSRC1/SORT1*	rs646776	109,620,053	C	0.21	0.83	0.79	0.87	4.3E-16	0.24	0.90	0.84	0.96	2.7E-03	0.85	0.82	0.88	3.1E-17
19	*LDLR/SMARCA4*	rs6511720	11,063,306	T	0.11	0.85	0.80	0.90	2.8E-08	0.08	0.91	0.81	1.01	8.5E-02	0.86	0.82	0.91	1.1E-08
19	*APOE/TOMM40*	rs2075650	50,087,459	G	0.14	1.14	1.08	1.20	1.0E-06	0.11	1.13	1.03	1.24	9.7E-03	1.14	1.09	1.19	3.2E-08
13	*COL4A1*	rs4773144	109,758,713	G	0.44	1.08	1.04	1.12	5.5E-05	0.42	1.13	1.06	1.20	7.2E-05	1.09	1.06	1.13	3.5E-08
10	*CYP17A1*	rs3824755	104,585,839	C	0.09	0.87	0.82	0.93	1.5E-05	0.24	0.90	0.84	0.96	1.8E-03	0.88	0.84	0.92	1.2E-07
7	*ZC3HC1*	rs11556924	129,450,732	T	0.38	0.92	0.88	0.95	7.6E-06	0.20	0.91	0.85	0.98	1.3E-02	0.92	0.89	0.95	3.1E-07
10	*LIPA*	rs2246942	90,994,866	G	0.34	1.09	1.05	1.14	4.9E-06	0.49	1.07	1.01	1.14	2.8E-02	1.09	1.05	1.12	4.8E-07
1	*MIA3*	rs2291832	220,893,104	G	0.27	0.91	0.87	0.95	1.0E-05	0.38	0.93	0.87	0.98	1.4E-02	0.92	0.88	0.95	4.9E-07
9	*ABO/SURF1/DBH**	rs651007	135,143,696	T	0.22	1.13	1.08	1.18	1.3E-07	0.17	1.02	0.95	1.11	5.5E-01	1.10	1.06	1.14	9.0E-07
20	*PROCR/EDEM2/MYH7B*	rs867186	33,228,215	G	0.10	0.85	0.80	0.90	1.9E-07	0.19	0.96	0.89	1.04	3.5E-01	0.89	0.85	0.94	3.5E-06
19	*CYP4F2*	rs2074901	15,858,422	C	0.17	1.09	1.04	1.15	3.4E-04	0.15	1.12	1.03	1.22	6.9E-03	1.10	1.05	1.15	8.3E-06
12	*C12orf30*	rs17696736	110,971,201	G	0.45	1.09	1.05	1.13	7.0E-06	0.11	1.03	0.94	1.13	5.5E-01	1.08	1.04	1.12	1.1E-05
15	*FURIN*	rs2071410	89,221,944	G	0.33	1.08	1.04	1.13	5.6E-05	0.24	1.06	0.99	1.14	7.5E-02	1.08	1.04	1.12	1.2E-05
16	*HPR/HP/TXNL4B*	rs2000999	70,665,594	A	0.20	1.11	1.06	1.16	1.1E-05	0.37	1.05	0.98	1.11	1.5E-01	1.09	1.05	1.13	1.2E-05
2	*HADHA*	rs4665319	26,278,087	A	0.21	0.92	0.88	0.96	2.6E-04	0.23	0.92	0.86	0.99	2.5E-02	0.92	0.89	0.96	1.8E-05
4	*REST*	rs3796529	57,492,171	T	0.19	1.11	1.06	1.17	1.6E-05	0.23	1.05	0.98	1.12	1.9E-01	1.09	1.05	1.14	1.9E-05
19	*APOE/TOMM40*	rs7412	50,103,919	T	<0.01	-	-	-	-	0.04	0.63	0.51	0.78	2.0E-05	0.63	0.51	0.78	2.0E-05
12	*ALDH2/SH2B3*	rs10744777	110,717,401	C	0.32	0.92	0.89	0.96	6.4E-05	0.66	0.95	0.89	1.01	9.9E-02	0.93	0.90	0.96	2.0E-05
15	*IGF1R*	rs7173377	97,306,251	C	0.40	1.06	1.02	1.10	4.4E-03	0.58	1.11	1.04	1.18	7.8E-04	1.07	1.04	1.11	2.5E-05
6	*APOM*	rs3130617	31,735,502	C	0.25	0.94	0.90	0.98	5.3E-03	0.21	0.87	0.81	0.94	3.8E-04	0.92	0.89	0.96	2.9E-05
7	*PARP12*	rs2269997	139,369,869	A	0.21	0.92	0.87	0.96	1.0E-03	0.16	0.87	0.78	0.96	8.9E-03	0.91	0.86	0.95	4.1E-05
8	*LPL*	rs331	19,864,685	A	0.27	0.93	0.89	0.97	3.7E-04	0.23	0.93	0.86	1.00	4.2E-02	0.93	0.89	0.96	4.1E-05
2	*IRS1*	rs2943634	226,776,324	A	0.33	0.93	0.89	0.96	1.1E-04	0.25	0.95	0.89	1.02	1.6E-01	0.93	0.90	0.96	5.0E-05
1	*C1orf21*	rs4631655	182,627,939	G	0.18	1.08	1.03	1.13	1.3E-03	0.17	1.11	1.02	1.20	1.2E-02	1.09	1.04	1.13	5.3E-05
3	*P2RY1*	rs1371097	154,034,880	T	0.15	1.07	1.02	1.13	5.5E-03	0.20	1.12	1.04	1.21	2.6E-03	1.09	1.04	1.14	6.6E-05
1	*TNNT2*	rs868407	199,607,964	C	0.28	1.07	1.03	1.11	1.0E-03	0.18	1.10	1.01	1.19	2.0E-02	1.08	1.04	1.11	6.7E-05
8	*LPL*	rs328	19,864,004	G	0.10	0.88	0.83	0.93	3.7E-05	0.09	0.96	0.86	1.06	4.2E-01	0.90	0.85	0.95	7.0E-05
6	*FOXC1*	rs2569881	1,565,036	A	0.14	0.90	0.85	0.95	1.2E-04	0.06	0.93	0.82	1.06	2.8E-01	0.90	0.86	0.95	7.1E-05
2	*ABCG8*	rs4299376	43,926,080	G	0.32	1.08	1.04	1.13	5.0E-05	0.27	1.03	0.96	1.10	3.6E-01	1.07	1.04	1.11	7.4E-05
5	*IL5*	rs2706399	131,895,601	A	0.49	0.94	0.90	0.97	4.3E-04	0.54	0.95	0.89	1.00	7.1E-02	0.94	0.91	0.97	8.0E-05
2	*MAT2A*	rs3755015	85,617,552	A	0.46	1.07	1.04	1.11	1.1E-04	0.40	1.03	0.97	1.10	2.8E-01	1.06	1.03	1.10	1.1E-04
1	*CASP9*	rs4646043	15,704,697	G	0.03	0.71	0.60	0.85	1.1E-04	<0.01	-	-	-	-	0.71	0.60	0.85	1.1E-04
17	*STAT5B*	rs17500235	37,609,886	G	0.06	1.15	1.07	1.24	2.7E-04	0.03	1.12	0.94	1.33	2.0E-01	1.14	1.07	1.22	1.2E-04
7	*TBXAS1*	rs757835	139,363,647	C	0.20	0.93	0.88	0.97	9.1E-04	0.16	0.92	0.85	1.00	5.3E-02	0.92	0.89	0.96	1.2E-04
1	*PMVK*	rs877343	153,176,636	G	0.43	1.08	1.04	1.12	5.5E-05	0.62	1.02	0.96	1.09	4.5E-01	1.06	1.03	1.10	1.2E-04
8	*TRIB1*	rs17321515	126,555,591	G	0.47	0.92	0.89	0.96	2.2E-05	0.36	0.99	0.93	1.05	7.5E-01	0.94	0.91	0.97	1.3E-04
7	*ATG9B*	rs3918226	150,321,109	T	0.09	1.13	1.06	1.21	4.0E-04	0.01	1.23	0.94	1.60	1.3E-01	1.14	1.06	1.21	1.4E-04
16	*NQO1*	rs4986998	68,306,370	A	0.04	1.14	1.04	1.25	5.7E-03	0.06	1.19	1.05	1.35	7.9E-03	1.16	1.07	1.25	1.4E-04
15	*IGF1R*	rs17847195	97,309,584	A	0.22	1.08	1.04	1.13	3.5E-04	0.32	1.05	0.99	1.12	1.3E-01	1.07	1.03	1.11	1.4E-04
4	*EDNRA*	rs6841473	148,627,102	T	0.14	1.06	1.01	1.12	2.5E-02	0.21	1.14	1.06	1.22	6.7E-04	1.09	1.04	1.13	1.5E-04
10	*TCF7L2*	rs12243326	114,778,805	C	0.28	1.07	1.02	1.11	2.1E-03	0.27	1.08	1.01	1.15	2.7E-02	1.07	1.03	1.11	1.6E-04
19	*RFX2*	rs1865028	5,966,066	C	0.01	1.24	1.05	1.45	1.1E-02	0.03	1.30	1.08	1.55	5.2E-03	1.26	1.12	1.42	1.7E-04
5	*RPS14*	rs2569107	149,802,040	T	0.04	1.12	1.01	1.23	2.4E-02	0.18	1.13	1.04	1.22	2.7E-03	1.12	1.06	1.19	1.7E-04
6	*HFE*	rs1800758	26,201,215	A	0.12	1.09	1.03	1.16	2.0E-03	0.22	1.08	1.01	1.16	3.2E-02	1.09	1.04	1.14	1.7E-04
17	*CDK5R1*	rs4132610	27,825,554	C	0.39	1.08	1.04	1.12	6.6E-05	0.48	1.02	0.96	1.08	5.3E-01	1.06	1.03	1.10	2.0E-04
2	*RBMS1*	rs6718526	160,922,421	T	0.20	0.92	0.88	0.96	2.3E-04	0.19	0.96	0.89	1.03	2.8E-01	0.93	0.89	0.97	2.1E-04
1	*AQP10*	rs1194610	152,562,700	C	0.23	0.93	0.89	0.97	9.4E-04	0.17	0.93	0.86	1.01	9.8E-02	0.93	0.90	0.97	2.2E-04
12	*TIMELESS*	rs11171846	55,114,323	T	0.09	1.13	1.06	1.20	7.8E-05	0.04	1.01	0.87	1.17	9.3E-01	1.11	1.05	1.18	2.3E-04
3	*TF*	rs8177191	134,950,829	A	0.17	0.92	0.88	0.97	1.0E-03	0.11	0.92	0.84	1.02	1.0E-01	0.92	0.88	0.96	2.4E-04
14	*VRK1*	rs1003229	97,241,370	G	0.23	1.08	1.03	1.13	5.2E-04	0.26	1.05	0.98	1.12	1.8E-01	1.07	1.03	1.11	2.7E-04
22	*OSM*	rs9608859	28,997,277	T	0.42	0.93	0.90	0.97	1.9E-04	0.27	0.97	0.91	1.04	4.3E-01	0.94	0.91	0.97	2.7E-04
2	*CXCR4*	rs7574456	136,606,529	C	0.23	0.95	0.91	0.99	2.3E-02	0.32	0.90	0.85	0.96	1.7E-03	0.94	0.90	0.97	2.7E-04
19	*PTGIR/PRKD2*	rs11083840	51,811,750	G	0.41	0.93	0.90	0.97	1.7E-04	0.50	0.98	0.92	1.04	4.1E-01	0.94	0.91	0.97	2.9E-04
1	*IL6R*	rs4845625	152,688,691	T	0.43	1.06	1.02	1.10	3.5E-03	0.43	1.07	1.01	1.13	3.2E-02	1.06	1.03	1.09	3.1E-04
5	*LOC91137*	rs313617	109,845,448	G	0.11	1.05	0.99	1.12	7.9E-02	0.25	1.13	1.06	1.21	4.8E-04	1.09	1.04	1.14	3.3E-04
6	*C2*	rs537160	32,024,379	A	0.32	0.93	0.90	0.97	6.4E-04	0.11	0.95	0.86	1.04	2.6E-01	0.94	0.90	0.97	3.4E-04
1	*MCL1*	rs11204666	148,809,752	T	0.16	0.95	0.90	1.00	3.5E-02	0.36	0.91	0.85	0.97	2.1E-03	0.93	0.90	0.97	3.6E-04
22	*CSF2RB*	rs11705394	35,659,622	C	0.25	1.07	1.02	1.11	2.8E-03	0.27	1.07	1.00	1.14	5.1E-02	1.07	1.03	1.11	3.6E-04
3	*NR1I2*	rs12721602	120,982,546	A	0.02	1.29	1.12	1.48	3.6E-04	<0.01	-	-	-	-	1.29	1.12	1.48	3.6E-04
3	*EIF4E3*	rs12629971	71,866,008	T	0.15	1.10	1.04	1.17	2.0E-03	0.13	1.11	0.99	1.25	7.4E-02	1.10	1.05	1.17	3.7E-04
3	*MST1*	rs6804655	49,713,949	T	0.31	1.07	1.03	1.11	1.1E-03	0.28	1.05	0.98	1.12	1.5E-01	1.06	1.03	1.10	3.8E-04
8	*CSMD3*	rs16883953	113,771,307	G	0.02	1.15	1.01	1.31	3.4E-02	0.04	1.27	1.09	1.49	2.6E-03	1.20	1.08	1.32	3.9E-04
5	*PDE4D*	rs2279737	58,370,402	G	0.33	1.05	1.01	1.10	8.1E-03	0.22	1.10	1.02	1.18	1.3E-02	1.06	1.03	1.10	4.5E-04
5	*ACOT12*	rs10942262	80,711,252	A	0.44	1.06	1.02	1.10	1.2E-03	0.49	1.04	0.98	1.11	1.6E-01	1.06	1.03	1.09	4.5E-04
3	*IL5RA*	rs4322988	3,095,571	T	0.12	1.08	1.02	1.14	7.7E-03	0.21	1.09	1.01	1.17	2.2E-02	1.08	1.04	1.13	4.6E-04
7	*C7orf10*	rs4320458	40,492,490	G	0.13	1.07	1.02	1.13	9.5E-03	0.19	1.10	1.02	1.18	1.7E-02	1.08	1.04	1.13	4.7E-04
11	*MMP20*	rs17098593	101,954,422	A	0.06	0.86	0.79	0.93	1.7E-04	0.04	0.98	0.84	1.13	7.4E-01	0.88	0.82	0.95	4.9E-04
21	*ABCG1*	rs532345	42,562,560	T	0.42	0.96	0.92	0.99	2.7E-02	0.31	0.91	0.85	0.97	2.4E-03	0.94	0.91	0.98	5.4E-04
2	*HDAC4*	rs3752755	239,669,182	T	0.36	1.07	1.03	1.11	1.1E-03	0.45	1.05	0.97	1.14	2.4E-01	1.06	1.03	1.10	5.4E-04
17	*PLCD3*	rs9303454	40,556,444	A	0.09	1.09	1.03	1.16	5.7E-03	0.06	1.15	1.02	1.31	2.8E-02	1.10	1.04	1.17	5.5E-04
7	*MAD1L1*	rs6948971	1,820,789	G	0.21	0.94	0.90	0.99	9.5E-03	0.40	0.93	0.88	0.99	2.2E-02	0.94	0.90	0.97	5.5E-04
2	*VAMP8*	rs1010	85,662,493	C	0.42	1.06	1.03	1.10	8.7E-04	0.38	1.04	0.97	1.10	2.5E-01	1.06	1.02	1.09	5.7E-04

Table S3 continued. Results for all loci meeting P<10^{-3} in discovery stage meta-analyses.

					European studies					South Asian studies					Combined studies			
chr	*Nearest gene(s)*	SNP	Position	Allele	Freq	OR	95% CI		P	Freq	OR	95% CI		P	OR	95% CI		P
10	*CUBN*	rs11254370	17,180,240	A	0.27	1.06	1.02	1.11	4.4E-03	0.42	1.06	1.00	1.13	5.3E-02	1.06	1.03	1.10	**5.7E-04**
1	*MFAP2*	rs2284746	17,179,262	C	0.48	1.04	1.01	1.08	2.3E-02	0.47	1.09	1.03	1.16	4.6E-03	1.06	1.02	1.09	**6.4E-04**
3	*PCCB*	rs9853387	137,521,678	G	0.34	1.07	1.03	1.11	**9.0E-04**	0.34	1.04	0.97	1.10	2.7E-01	1.06	1.02	1.09	**6.5E-04**
14	*ESR2*	rs1256114	63,879,758	A	0.13	0.94	0.89	0.99	2.0E-02	0.09	0.86	0.77	0.95	3.8E-03	0.92	0.88	0.97	**6.8E-04**
6	*C4B*	rs389883	32,055,439	G	0.28	0.93	0.89	0.97	**5.5E-04**	0.11	0.97	0.88	1.07	5.8E-01	0.94	0.90	0.97	**6.8E-04**
11	*PDGFD*	rs2129741	103,373,185	C	0.38	1.07	1.03	1.12	**2.4E-04**	0.35	1.02	0.95	1.08	6.3E-01	1.06	1.02	1.09	**7.0E-04**
2	*APOB*	rs12714264	21,119,023	T	0.13	0.93	0.88	0.99	1.4E-02	0.13	0.89	0.82	0.98	1.3E-02	0.92	0.88	0.97	**7.0E-04**
8	*NRG1*	rs17665441	32,574,569	T	0.48	0.94	0.91	0.98	2.5E-03	0.48	0.95	0.90	1.01	1.3E-01	0.95	0.92	0.98	**7.3E-04**
17	*GAS7*	rs4572442	9,977,521	G	0.43	1.07	1.03	1.11	**5.3E-04**	0.30	1.02	0.96	1.09	4.7E-01	1.06	1.02	1.09	**7.4E-04**
4	*NFKB1*	rs4647972	103,649,564	T	0.04	0.86	0.78	0.96	5.2E-03	0.03	0.85	0.72	1.01	5.8E-02	0.86	0.79	0.94	**7.4E-04**
12	*HMGA2*	rs12423095	64,577,499	C	0.01	1.11	0.95	1.30	1.9E-01	0.21	1.12	1.04	1.21	1.9E-03	1.12	1.05	1.20	**7.5E-04**
17	*RPS6KB1*	rs1296279	55,320,513	A	0.19	1.10	1.05	1.15	**7.1E-05**	0.27	1.01	0.94	1.08	8.7E-01	1.07	1.03	1.11	**7.5E-04**
12	*P2RX4*	rs10849859	120,151,810	T	0.11	0.91	0.86	0.97	1.9E-03	0.16	0.94	0.87	1.02	1.5E-01	0.92	0.88	0.97	**7.5E-04**
2	*ITGA4*	rs1449264	182,044,719	C	0.34	0.94	0.90	0.98	2.9E-03	0.25	0.95	0.88	1.01	1.1E-01	0.94	0.91	0.97	**7.6E-04**
4	*ADD1*	rs3775068	2,858,239	A	0.44	1.07	1.03	1.11	**5.4E-04**	0.34	1.02	0.96	1.09	4.6E-01	1.06	1.02	1.09	**7.8E-04**
10	*ALOX5*	rs3780909	45,245,221	T	0.49	0.94	0.90	0.97	**5.9E-04**	0.42	0.98	0.92	1.04	4.4E-01	0.95	0.92	0.98	**8.2E-04**
17	*SERPINF1*	rs1136287	1,620,026	C	0.35	1.04	1.01	1.09	2.7E-02	0.50	1.09	1.02	1.15	6.9E-03	1.06	1.02	1.09	**8.7E-04**
20	*CYP24A1*	rs2245153	52,219,813	C	0.20	0.93	0.89	0.97	2.4E-03	0.21	0.95	0.88	1.02	1.6E-01	0.94	0.90	0.97	**8.8E-04**
6	*TAP2*	rs9357155	32,917,826	A	0.12	1.07	1.01	1.14	1.6E-02	0.14	1.11	1.02	1.21	1.7E-02	1.08	1.03	1.14	**8.9E-04**
10	*NRG3*	rs17099655	83,993,667	A	0.15	1.06	1.01	1.11	2.8E-02	0.11	1.15	1.05	1.27	3.4E-03	1.08	1.03	1.13	**9.0E-04**
19	*TGFB1*	rs2241714	46,561,232	T	0.32	1.06	1.02	1.10	5.8E-03	0.39	1.06	1.00	1.13	6.6E-02	1.06	1.02	1.09	**9.2E-04**
3	*P2RY1*	rs16864605	154,033,761	C	0.04	1.17	1.07	1.29	**7.1E-04**	0.10	1.06	0.96	1.17	2.2E-01	1.12	1.05	1.20	**9.4E-04**
6	*ENPP1*	rs6935458	132,168,013	G	0.10	0.94	0.88	1.00	5.8E-02	0.12	0.86	0.79	0.95	2.0E-03	0.92	0.87	0.97	**9.5E-04**
1	*PCSK9*	rs10888896	55,281,801	G	0.26	1.05	1.01	1.10	1.4E-02	0.19	1.10	1.02	1.18	1.8E-02	1.06	1.03	1.10	**9.6E-04**
10	*CTNNA3*	rs10509281	68,641,096	G	0.03	1.16	1.05	1.29	4.5E-03	0.09	1.10	0.99	1.22	6.9E-02	1.13	1.05	1.22	**9.9E-04**
10	*VCL*	rs3793921	75,538,120	C	0.25	0.96	0.92	1.00	4.3E-02	0.43	0.92	0.86	0.97	4.2E-03	0.94	0.91	0.98	**9.9E-04**
11	*TUB*	rs10839972	8,032,750	T	0.20	1.08	1.03	1.13	1.8E-03	0.15	1.05	0.97	1.14	2.5E-01	1.07	1.03	1.11	**9.9E-04**
20	*ACSS2*	rs2295097	32,941,882	C	0.07	0.89	0.83	0.96	2.1E-03	0.08	0.93	0.83	1.04	1.8E-01	0.90	0.85	0.96	1.0E-03
6	*VEGFA*	rs9472138	43,919,740	T	0.29	0.96	0.91	1.00	6.7E-02	0.21	0.83	0.75	0.92	**2.0E-04**	0.93	0.89	0.97	1.0E-03
15	*AKAP13*	rs7177107	83,924,368	A	0.22	1.10	1.04	1.16	**5.6E-04**	0.12	1.02	0.90	1.15	7.7E-01	1.08	1.03	1.14	1.0E-03
20	*MMP24*	rs2275274	33,320,958	T	0.09	0.90	0.84	0.96	**9.5E-04**	0.19	0.96	0.89	1.03	2.5E-01	0.92	0.88	0.97	1.1E-03
17	*SERPINF1*	rs2071021	1,626,543	G	0.29	1.04	0.99	1.08	1.2E-01	0.33	1.12	1.05	1.19	**6.5E-04**	1.06	1.02	1.10	1.1E-03
1	*LMOD1*	rs4494199	200,151,291	G	0.45	0.93	0.90	0.97	**2.9E-04**	0.38	0.99	0.93	1.06	8.3E-01	0.95	0.92	0.98	1.3E-03
5	*F2RL1*	rs2243060	76,164,123	A	0.21	1.17	1.07	1.28	**7.7E-04**	0.30	1.05	0.99	1.12	1.2E-01	1.09	1.03	1.15	1.3E-03
11	*SMPD1*	rs11040883	6,366,366	A	0.15	0.91	0.86	0.95	**1.8E-04**	0.15	1.00	0.92	1.09	9.8E-01	0.93	0.89	0.97	1.3E-03
19	*PLAUR*	rs4760	48,844,940	G	0.16	1.10	1.05	1.16	**1.9E-04**	0.14	1.00	0.91	1.09	9.5E-01	1.07	1.03	1.12	1.4E-03
17	*PLCD3*	rs7207047	40,554,524	A	0.41	0.93	0.90	0.97	**1.6E-04**	0.31	1.01	0.94	1.07	8.5E-01	0.95	0.92	0.98	1.5E-03
17	*MAP2K4*	rs9903561	11,989,760	A	0.15	0.90	0.85	0.96	**6.4E-04**	0.08	1.00	0.86	1.16	9.9E-01	0.91	0.86	0.97	1.5E-03
2	*CXCR4*	rs10191360	136,601,149	T	0.45	0.97	0.93	1.00	8.7E-02	0.34	0.90	0.84	0.96	**8.2E-04**	0.95	0.92	0.98	1.6E-03
17	*MAP2K6*	rs8077944	64,993,948	A	0.47	1.06	1.03	1.10	**9.7E-04**	0.46	1.02	0.96	1.08	5.1E-01	1.05	1.02	1.08	1.6E-03
3	*CHRD*	rs885838	185,579,272	A	0.49	1.06	1.03	1.10	**8.0E-04**	0.44	1.02	0.96	1.08	5.8E-01	1.05	1.02	1.08	1.7E-03
14	*TGFB3*	rs3917148	75,516,274	G	0.08	1.14	1.06	1.22	**1.7E-04**	0.10	1.00	0.91	1.10	9.6E-01	1.09	1.03	1.15	1.8E-03
1	*APCS*	rs2166587	157,816,617	T	0.09	1.12	1.05	1.19	**8.8E-04**	0.09	1.03	0.93	1.14	5.9E-01	1.09	1.03	1.15	1.9E-03
4	*ARD1B*	rs9790513	80,305,472	A	0.18	1.09	1.04	1.14	**2.9E-04**	0.13	0.99	0.90	1.08	7.7E-01	1.07	1.02	1.11	2.2E-03
3	*CAMK1*	rs293782	9,794,922	C	0.18	0.92	0.87	0.96	**2.8E-04**	0.14	1.01	0.93	1.10	7.7E-01	0.94	0.90	0.98	2.4E-03
20	*GNAS*	rs6026567	56,878,310	G	0.42	1.06	1.03	1.10	**9.3E-04**	0.41	1.01	0.95	1.07	7.1E-01	1.05	1.02	1.08	2.6E-03
4	*KDR*	rs17085262	55,653,898	T	0.24	0.97	0.93	1.01	1.2E-01	0.15	0.86	0.79	0.93	**3.8E-04**	0.94	0.91	0.98	2.7E-03
6	*LTA/TNF*	rs1800629	31,651,010	A	0.17	0.92	0.88	0.97	**9.1E-04**	0.08	1.01	0.90	1.12	9.2E-01	0.93	0.89	0.98	2.8E-03
11	*SORL1*	rs9633951	121,000,351	C	0.44	1.07	1.03	1.11	**8.3E-04**	0.43	1.01	0.95	1.07	8.3E-01	1.05	1.02	1.08	3.1E-03
6	*MICB/MICA/BAT1*	rs3095235	31,571,149	G	0.17	0.96	0.92	1.01	1.5E-01	0.06	0.76	0.67	0.86	**2.4E-05**	0.94	0.89	0.98	4.1E-03
19	*XRCC1*	rs1799782	48,749,414	A	0.06	0.96	0.89	1.04	3.4E-01	0.08	0.82	0.74	0.92	**3.8E-04**	0.91	0.86	0.97	4.6E-03
8	*CSMD3*	rs2883875	114,253,595	G	0.46	0.98	0.94	1.01	2.0E-01	0.35	0.90	0.84	0.96	**6.8E-04**	0.96	0.93	0.99	4.8E-03
17	*TRPV1*	rs16953301	3,460,096	T	0.03	1.19	1.08	1.31	**5.4E-04**	0.03	0.95	0.79	1.14	5.8E-01	1.13	1.04	1.23	5.5E-03
1	*PCSK9*	rs7552841	55,291,340	T	0.38	1.08	1.04	1.12	**7.9E-05**	0.32	0.96	0.90	1.02	2.2E-01	1.05	1.01	1.08	5.6E-03
22	*PDGFB*	rs4821874	37,956,321	A	0.27	1.07	1.03	1.12	**8.5E-04**	0.33	1.00	0.94	1.06	9.4E-01	1.05	1.01	1.09	5.8E-03
15	*ISG20*	rs4566136	86,981,525	C	0.49	0.94	0.91	0.97	**8.6E-04**	0.46	1.01	0.95	1.07	8.1E-01	0.96	0.93	0.99	6.5E-03
1	*IL6R*	rs4509570	152,703,008	G	0.24	1.03	0.98	1.07	2.6E-01	0.24	1.13	1.05	1.21	**8.1E-04**	1.05	1.01	1.09	6.6E-03
2	*ABCB11*	rs484066	169,490,727	A	0.37	0.94	0.90	0.97	**5.9E-04**	0.31	1.02	0.95	1.09	5.7E-01	0.96	0.93	0.99	7.3E-03
12	*PDE3A*	rs10743366	20,417,893	G	0.44	1.08	1.03	1.12	**5.5E-04**	0.45	0.99	0.93	1.05	7.6E-01	1.05	1.01	1.08	7.5E-03
1	*TNRC4*	rs2280474	149,948,191	A	0.13	0.91	0.86	0.96	**5.1E-04**	0.09	1.05	0.94	1.16	4.0E-01	0.94	0.89	0.98	7.7E-03
2	*LCT*	rs2322659	136,272,129	T	0.26	1.00	0.95	1.06	9.9E-01	0.52	1.13	1.06	1.19	**9.8E-05**	1.06	1.01	1.10	8.6E-03
10	*GRK5*	rs291979	121,119,787	A	0.23	0.98	0.94	1.03	4.0E-01	0.22	0.87	0.81	0.94	**2.3E-04**	0.95	0.92	0.99	8.9E-03
12	*BCAT1*	rs11047689	24,933,171	C	0.18	1.13	1.07	1.19	**2.2E-06**	0.20	0.92	0.85	0.99	2.4E-02	1.06	1.01	1.10	9.0E-03
22	*BID*	rs181410	16,617,436	T	0.36	0.98	0.95	1.02	4.1E-01	0.26	0.87	0.82	0.94	**1.2E-04**	0.96	0.93	0.99	9.4E-03
19	*TGFB1*	rs4803455	46,543,349	A	0.49	0.98	0.95	1.02	3.3E-01	0.46	0.90	0.85	0.96	**9.2E-04**	0.96	0.93	0.99	1.0E-02
9	*TRIM32*	rs3019	118,501,933	C	0.09	1.12	1.05	1.20	**3.9E-04**	0.07	0.94	0.83	1.05	2.6E-01	1.08	1.02	1.14	1.1E-02
19	*GP6*	rs2304167	60,218,893	C	0.18	0.98	0.94	1.03	4.6E-01	0.27	0.89	0.83	0.96	**9.7E-04**	0.95	0.91	0.99	1.2E-02
8	*ADRA1A*	rs1390514	26,732,950	C	0.02	1.26	1.10	1.45	**9.2E-04**	0.04	1.00	0.85	1.17	9.7E-01	1.14	1.03	1.27	1.2E-02
8	*MMP16*	rs16881203	89,412,574	T	0.21	0.99	0.94	1.03	5.7E-01	0.27	0.88	0.83	0.94	**2.9E-04**	0.95	0.92	0.99	1.3E-02
3	*CASR*	rs1042636	123,486,459	G	0.07	0.89	0.83	0.95	**8.0E-04**	0.20	1.00	0.93	1.08	9.6E-01	0.94	0.89	0.99	1.3E-02
13	*EDNRB*	rs3818416	77,372,469	A	0.23	1.08	1.03	1.13	**6.8E-04**	0.21	0.97	0.90	1.04	3.7E-01	1.05	1.01	1.09	1.4E-02
11	*TUB*	rs11041718	8,011,844	G	0.47	1.00	0.97	1.04	9.3E-01	0.43	1.15	1.08	1.22	**1.1E-05**	1.04	1.01	1.07	1.8E-02
6	*EDN1*	rs1626492	12,403,489	A	0.28	0.98	0.94	1.03	4.3E-01	0.17	0.87	0.81	0.95	**7.9E-04**	0.95	0.92	0.99	1.9E-02
20	*NFATC2*	rs228840	49,504,665	A	0.16	0.91	0.87	0.96	**5.4E-04**	0.17	1.05	0.97	1.14	2.2E-01	0.95	0.91	0.99	2.5E-02
1	*ADORA3*	rs13376212	111,905,003	A	0.08	1.13	1.05	1.21	**8.3E-04**	0.12	0.97	0.88	1.06	5.0E-01	1.07	1.01	1.13	2.5E-02

Table S3 continued. Results for all loci meeting $P<10^{-3}$ in discovery stage meta-analyses.

					European studies					South Asian studies					Combined studies			
**chr**	***Nearest gene(s)***	**SNP**	**Position**	**Allele**	**Freq**	**OR**	**95% CI**		**P**	**Freq**	**OR**	**95% CI**		**P**	**OR**	**95% CI**		**P**
1	*PPAP2B*	rs12038246	56,842,202	A	0.49	1.08	1.03	1.13	**5.1E-04**	0.42	0.93	0.86	1.01	7.0E-02	1.04	1.00	1.08	2.8E-02
8	*FDFT1*	rs1293314	11,727,515	T	0.04	0.98	0.89	1.08	7.1E-01	0.05	0.79	0.68	0.91	**8.3E-04**	0.92	0.85	0.99	3.0E-02
14	*ESR2*	rs1255998	63,763,624	C	0.10	1.00	0.94	1.06	8.9E-01	0.15	0.86	0.79	0.94	**5.9E-04**	0.95	0.90	1.00	3.1E-02
4	*UCP1*	rs6822807	141,707,198	C	0.26	1.08	1.04	1.13	**2.3E-04**	0.32	0.95	0.89	1.01	9.0E-02	1.04	1.00	1.08	3.2E-02
20	*NFATC2*	rs12624399	49,500,622	C	0.04	0.96	0.86	1.07	4.2E-01	0.13	1.17	1.07	1.28	**5.7E-04**	1.08	1.01	1.15	3.3E-02
18	*BCL2*	rs2046135	58,965,232	A	0.03	1.19	1.08	1.32	**7.9E-04**	0.10	0.98	0.89	1.09	7.1E-01	1.08	1.00	1.16	3.6E-02
8	*MSRA*	rs13249013	10,042,777	G	0.34	1.07	1.03	1.11	**4.4E-04**	0.48	0.95	0.89	1.01	8.7E-02	1.03	1.00	1.07	4.3E-02
7	*SRI*	rs173850	87,681,063	A	0.43	0.99	0.96	1.03	7.0E-01	0.31	0.90	0.84	0.96	**8.9E-04**	0.97	0.94	1.00	4.8E-02
19	*IL11*	rs2305788	60,582,646	G	0.06	1.14	1.06	1.23	**6.2E-04**	0.10	0.94	0.85	1.03	1.9E-01	1.06	1.00	1.13	4.9E-02
1	*FMOD*	rs3820224	201,579,179	T	0.12	0.91	0.86	0.96	**9.2E-04**	0.15	1.06	0.97	1.15	1.8E-01	0.95	0.91	1.00	4.9E-02
17	*GLP2R*	rs17681684	9,733,493	A	0.30	1.01	0.97	1.05	5.3E-01	0.13	1.16	1.06	1.27	**9.2E-04**	1.04	1.00	1.08	5.2E-02
15	*ADAMTS7*	rs11631955	76,872,970	G	0.43	1.00	0.95	1.05	9.9E-01	0.32	0.85	0.78	0.93	**1.8E-04**	0.96	0.92	1.00	5.3E-02
12	*PDE3A*	rs7137534	20,723,044	T	0.32	1.00	0.96	1.04	8.9E-01	0.29	0.89	0.84	0.95	**7.1E-04**	0.97	0.94	1.00	6.5E-02
2	*PDK1*	rs16860693	173,161,524	C	0.03	0.95	0.84	1.08	4.6E-01	0.08	1.32	1.14	1.53	**2.7E-04**	1.10	0.99	1.21	6.6E-02
12	*VWF*	rs11064024	6,072,310	G	0.37	1.07	1.03	1.12	**6.1E-04**	0.30	0.93	0.87	0.99	3.1E-02	1.03	1.00	1.07	7.6E-02
8	*NCALD*	rs9642976	103,185,175	G	0.15	1.00	0.93	1.08	9.8E-01	0.11	1.24	1.10	1.41	**7.1E-04**	1.06	0.99	1.13	7.8E-02
8	*SDC2*	rs724235	97,652,163	A	0.25	1.00	0.96	1.04	9.0E-01	0.30	1.12	1.05	1.19	**7.4E-04**	1.03	1.00	1.07	7.9E-02
10	*PDE6C*	rs1223306	95,370,780	G	0.48	0.99	0.95	1.03	5.9E-01	0.40	1.13	1.06	1.20	**1.5E-04**	1.02	0.99	1.06	1.4E-01
15	*NRG4*	rs17428804	74,047,485	T	0.11	1.01	0.95	1.07	8.3E-01	0.06	0.78	0.68	0.89	**1.7E-04**	0.97	0.92	1.02	1.9E-01
5	*GPR98*	rs13158963	90,484,910	A	0.11	1.03	0.97	1.09	3.9E-01	0.18	0.88	0.81	0.95	**9.0E-04**	0.97	0.93	1.02	1.9E-01
13	*EFNB2*	rs913607	105,708,805	G	0.20	1.03	0.98	1.07	2.9E-01	0.40	0.90	0.85	0.96	**8.3E-04**	0.98	0.94	1.01	2.4E-01
3	*CISH*	rs873985	50,626,755	G	0.16	0.98	0.93	1.03	3.8E-01	0.17	1.15	1.06	1.24	**5.8E-04**	1.02	0.98	1.07	2.6E-01
3	*CASR*	rs10934578	123,459,972	T	0.33	0.98	0.93	1.03	3.7E-01	0.49	1.15	1.06	1.25	**5.8E-04**	1.02	0.98	1.06	3.4E-01
1	*CPT2*	rs11578832	53,447,130	A	0.22	1.02	0.98	1.07	3.8E-01	0.10	0.84	0.76	0.93	**5.2E-04**	0.99	0.95	1.03	5.3E-01
1	*SELP/F5*	rs3917793	167,834,169	A	0.10	1.03	0.97	1.10	3.2E-01	0.05	0.78	0.68	0.90	**4.7E-04**	0.99	0.93	1.04	6.0E-01
18	*NFATC1*	rs1017860	75,273,194	T	0.19	0.97	0.92	1.01	1.5E-01	0.16	1.15	1.06	1.25	**6.3E-04**	1.01	0.97	1.05	6.7E-01
17	*HNF1B*	rs4430796	33,172,153	G	0.49	1.04	1.00	1.08	3.5E-02	0.38	0.89	0.84	0.95	**3.8E-04**	1.00	0.97	1.03	9.6E-01

SNPs are ordered by ascending P value in the combined meta-analysis. Only the lead SNP (with the lowest P value) from each locus is shown unless different SNPs met the threshold in Europeans/South Asians.

Data shown are per-allele odds ratios from unadjusted fixed-effect inverse-variance meta-analysis of 10 European studies, 2 South Asians studies and 12 studies combined.

Loci highlighted in grey are those previously identified by GWA studies; loci highlighted in yellow are additional loci considered to be known CAD risk loci.
