# Supplementary material for: Large-Scale Gene-Centric Analysis Identifies Novel Variants for Coronary Artery Disease
Source: PLoS Genet. 2011 Sep 22;7(9):e1002260. doi: 10.1371/journal.pgen.1002260 (PMC3178591; doi:10.1371/journal.pgen.1002260)
Supplement: Table S4 — Associations with previously studied candidate variants. Variants ordered by biological pathway, then gene. Per-allele odds ratios are presented for the effect allele, which is the minor allele in European populations. r2 with best imputed proxy was estimated using ∼2.5 M directly genotyped or HapMap-imputed SNPs in the CARDIoGRAM Consortium. Tagging levels are 1 (r2>0.8 with all HapMap/Seattle SNPs of MAF≥0.02), 2 (r2>0.5 with all HapMap/Seattle SNPs of MAF≥0.05), 3 (only non-synonymous and known functional variants of MAF>0.01) and GWAS (specific SNPs previously identified in recent GWAS). a rs4343 has r2 = 1 with the insertion/deletion polymorphism in the ACE gene in CEU HapMap 2 population. b rs17443251 has r2 = 0.75 with the more commonly studied R144C variant (rs1799853) in the CYP2C9 gene in CEU HapMap 2 population. c rs9526246 has r2 = 0.97 with the more commonly studied T102C variant (rs6313) in the HTR2A gene in CEU HapMap 2 population. d rs1062535 has r2 = 0.97 with the more commonly studied C807T variant (rs1126643) in the ITGA2 gene in CEU HapMap 2 population. e rs1805096 has r2 = 0.89 with the more commonly studied rs6700896 variant in the LEPR gene in CEU HapMap 2 population. f rs1049897 has r2 = 1 with the more commonly studied A102T variant (rs4236) in the MGP gene in CEU HapMap 2 population. g rs4968624 has r2 = 0.97 with the more commonly studied L125V variant (rs668) in the PECAM1 gene in CEU HapMap 2 population. h rs12944077 has r2 = 1 with the more commonly studied S563N variant (rs12953) in the PECAM1 gene in CEU HapMap 2 population. (PDF) [file pgen.1002260.s008.pdf]

Table S4. Associations with previously studied candidate variants.

Pathway	Locus	Gene	SNP	Variant	Position	Allele	Europeans Freq	OR	95% CI	P	South Asians Freq	OR	95% CI	P	r^2 with best imputed proxy	Tagging level
Cellular adhesion																
	19p13.2	*ICAM1*	rs5498	A649G	10,256,683	G	0.43	0.98	(0.94-1.02)	2.8E-01	0.49	1.05	(0.99-1.11)	1.2E-01	1	1
	17q23.3	*PECAM1*	rs4968624[g]		59,783,283	A	0.49	1.02	(0.98-1.05)	4.0E-01	0.43	1.00	(0.94-1.06)	9.9E-01	1	2
	17q23.3	*PECAM1*	rs12944077[h]		59,784,458	T	0.49	0.99	(0.95-1.02)	4.7E-01	0.43	1.00	(0.94-1.06)	9.8E-01	1	2
	1q24.2	*SELE*	rs5355	L554F	167,962,494	A	0.04	1.13	(1.03-1.25)	9.9E-03	0.08	1.03	(0.92-1.14)	6.1E-01	1	1
	1q24.2	*SELE*	rs5361	S128R	167,967,684	G	0.11	1.00	(0.94-1.06)	8.8E-01	0.08	1.00	(0.90-1.12)	9.9E-01	1	1
	1q24.2	*SELP*	rs6136	T715P	167,830,575	G	0.11	0.99	(0.94-1.05)	7.9E-01	0.04	0.87	(0.75-1.01)	7.2E-02	1	2
Lipids																
	9q31.3	*ABCA1*	rs2422943	C-477T	106,730,816	A	0.47	1.00	(0.96-1.04)	9.2E-01	0.49	1.07	(1.01-1.13)	3.1E-02	1	1
	9q31.3	*ABCA1*	rs2230806	R219K	106,660,688	T	0.27	0.98	(0.94-1.02)	4.0E-01	0.37	0.93	(0.87-0.99)	2.3E-02	1	1
	11q23.3	*APOA5*	rs662799	T-1131C	116,168,917	G	0.07	1.11	(1.03-1.19)	6.6E-03	0.18	1.02	(0.94-1.10)	6.9E-01	1	1
	2p24.1	*APOB*	rs1042031	EcoRI	21,079,258	T	0.18	0.99	(0.95-1.04)	7.5E-01	0.10	1.07	(0.97-1.18)	1.9E-01	1	1
	2p24.1	*APOB*	rs693	XBaI	21,085,700	G	0.49	1.00	(0.97-1.04)	8.6E-01	0.29	0.93	(0.87-1.00)	3.5E-02	1	1
	19q13.32	*APOE*	rs7412	R158C	50,103,919	T	<0.01	-	-	-	0.04	0.63	(0.51-0.78)	2.0E-05	0.59	1
	19q13.32	*APOE*	rs2075650		50,087,459	G	0.14	1.14	(1.08-1.20)	1.0E-06	0.11	1.13	(1.03-1.24)	9.7E-03	1	1
	1p13.3	*CELSR2*	rs646776		109,620,053	T	0.79	1.20	(1.15-1.27)	4.3E-16	0.76	1.11	(1.04-1.19)	2.7E-03	1	GWAS
	16q13	*CETP*	rs1800775	C-629A	55,552,737	A	0.48	1.00	(0.96-1.03)	8.5E-01	0.39	1.00	(0.94-1.06)	9.6E-01	1	1
	16q13	*CETP*	rs708272	TaqIB	55,553,789	A	0.42	1.00	(0.97-1.04)	8.4E-01	0.48	0.99	(0.94-1.06)	8.7E-01	0.97	1
	19p13.2	*LDLR*	rs2228671	C27C	11,071,912	T	0.12	0.89	(0.84-0.94)	2.7E-05	0.07	0.91	(0.81-1.02)	1.1E-01	1	1
	15q22.1	*LIPC*	rs1800588	C-514T	56,510,967	T	0.21	1.02	(0.98-1.07)	3.2E-01	0.24	0.93	(0.87-1.00)	3.6E-02	1	1
	6q26	*LPA*	rs10455872		160,930,108	G	0.07	1.45	(1.34-1.57)	1.5E-20	0.01	1.23	(0.84-1.78)	2.8E-01	1	1
	6q26	*LPA*	rs3798220	I4399M	160,881,127	C	0.02	1.74	(1.52-1.99)	1.0E-15	<0.01	-	-	-	-	1
	8p21.3	*LPL*	rs328	S447X	19,864,004	G	0.10	0.88	(0.83-0.93)	3.7E-05	0.09	0.96	(0.86-1.06)	4.2E-01	1	1
	4q23	*MTTP*	rs1800591	G-493T	100,714,511	T	0.26	0.99	(0.95-1.03)	6.9E-01	0.32	0.96	(0.91-1.03)	2.7E-01	1	1
	1p32.3	*PCSK9*	rs10888896		55,281,801	G	0.26	1.05	(1.01-1.10)	1.4E-02	0.19	1.10	(1.02-1.18)	1.8E-02	1	1
	1p32.3	*PCSK9*	rs11591147		55,278,235	G	0.99	1.25	(1.08-1.45)	3.7E-03	>0.99	-	-	-	-	1
	7q21.3	*PON1*	rs662	Q192R	94,775,382	C	0.29	0.97	(0.93-1.01)	1.1E-01	0.37	0.98	(0.92-1.05)	5.7E-01	1	1
	7q21.3	*PON2*	rs7493	S311C	94,872,711	C	0.23	0.96	(0.92-1.00)	6.9E-02	0.36	0.99	(0.93-1.05)	7.7E-01	1	2
	1q31.1	*PTGS2*	rs20417	G-765C	184,916,944	G	0.16	0.97	(0.92-1.02)	2.4E-01	0.16	1.08	(1.00-1.18)	4.8E-02	1	1
Inflammation/immunity																
	13q12.3	*ALOX5AP*	rs10507391	SG13S114	30,210,096	A	0.34	1.03	(0.97-1.09)	3.6E-01	0.39	0.96	(0.88-1.04)	3.2E-01	1	1
	17q12	*CCL11*	rs3744508	T23A	29,637,007	A	0.18	1.02	(0.96-1.08)	5.1E-01	0.16	0.90	(0.83-0.98)	1.1E-02	1	2
	3p21.31	*CCR2*	rs1799864	V64I	46,374,212	A	0.09	0.95	(0.86-1.04)	2.3E-01	0.10	0.87	(0.76-0.99)	3.1E-02	-	1
	5q31.3	*CD14*	rs2569190	C-159T	139,993,100	A	0.47	0.94	(0.87-1.02)	1.5E-01	0.45	0.94	(0.85-1.03)	1.6E-01	1	1
	1q23.2	*CRP*	rs1205	C2042T	157,948,857	T	0.33	1.01	(0.97-1.05)	5.9E-01	0.29	1.06	(0.99-1.13)	1.1E-01	1	1
	3p22.2	*CX3CR1*	rs3732378	T280M	39,282,166	A	0.17	0.99	(0.94-1.04)	5.6E-01	0.11	0.96	(0.87-1.06)	4.4E-01	1	2
	3p22.2	*CX3CR1*	rs3732379	V249I	39,282,260	T	0.28	1.00	(0.96-1.04)	9.2E-01	0.14	0.95	(0.87-1.03)	2.1E-01	1	2
	10q11.21	*CXCL12*	rs501120		44,073,873	T	0.86	1.08	(1.01-1.14)	3.3E-02	0.67	1.03	(0.95-1.12)	4.7E-01	1	2
	4q32.1	*FGB*	rs1800787	C-148T	155,703,465	T	0.21	1.03	(0.98-1.07)	2.8E-01	0.17	1.01	(0.94-1.10)	7.2E-01	1	1
	4q32.1	*FGB*	rs1800790	G-455A	155,703,158	A	0.20	1.04	(0.99-1.09)	1.5E-01	0.17	1.02	(0.94-1.10)	6.6E-01	1	1
	2q13	*IL1B*	rs16944	C-511T	113,311,338	A	0.33	1.00	(0.97-1.04)	8.6E-01	0.41	0.96	(0.90-1.02)	1.6E-01	1	1
	7p15.3	*IL6*	rs1800795	G-174C	22,733,170	C	0.42	1.02	(0.99-1.06)	2.3E-01	0.19	1.02	(0.95-1.10)	5.5E-01	1	1
	1q21.3	*IL6R*	rs4537545	-	152,685,503	T	0.41	0.96	(0.92-0.99)	1.8E-02	0.30	0.95	(0.89-1.01)	1.0E-01	1	1
	6p21.3	*LTA*	rs909253	A252G	31,648,292	G	0.34	0.99	(0.95-1.03)	5.8E-01	0.28	0.94	(0.88-1.00)	6.2E-02	1	1
	14q13.2	*PSMA6*	rs1048990	C-8G	34,831,426	G	0.17	1.04	(0.98-1.11)	1.7E-01	0.21	1.02	(0.95-1.10)	5.4E-01	1	2
	15q14	*THBS1*	rs2228262	N700S	37,669,470	G	0.11	1.00	(0.94-1.07)	9.9E-01	0.06	1.04	(0.92-1.18)	5.2E-01	1	2
	6q27	*THBS2*	rs8089	T3949G	169,359,651	C	0.25	1.02	(0.98-1.07)	2.9E-01	0.11	0.97	(0.89-1.07)	5.9E-01	1	2
	5q14.1	*THBS4*	rs1866389	A387P	79,397,021	C	0.22	1.02	(0.98-1.07)	2.8E-01	0.19	0.95	(0.88-1.03)	1.9E-01	1	2
	9q33.1	*TLR4*	rs4986790	D299G	119,515,123	G	0.06	1.02	(0.94-1.11)	5.9E-01	0.10	1.12	(1.01-1.24)	2.5E-02	1	1
	6p21.3	*TNF*	rs1800629	G-308A	31,651,010	A	0.17	0.92	(0.88-0.97)	9.1E-04	0.08	1.01	(0.90-1.12)	9.2E-01 #	1	1
	12p13.31	*TNFRSF1A*	rs4149584	R92Q	6,312,904	T	0.02	1.09	(0.95-1.25)	2.0E-01	<0.01	-	-	-	0.19	1

Table S4 continued. Associations with previously studied candidate variants.

Pathway	Locus	Gene	SNP	Variant	Position	Allele	Europeans Freq	OR	95% CI	P	South Asians Freq	OR	95% CI	P	r^2 with best imputed proxy	Tagging level
Hemostasis																
	5q35.3	*F12*	rs1801020	C46T	176,769,138	A	0.24	1.07	(1.02-1.12)	2.0E-03	0.42	1.02	(0.96-1.08)	5.4E-01	1	1
	6p25.1	*F13A1*	rs5985	V34L	6,263,794	A	0.25	0.98	(0.94-1.02)	3.8E-01	0.12	0.98	(0.90-1.08)	7.4E-01	1	2
	1q24.2	*F5*	rs6025	R506Q	167,785,673	T	0.03	0.90	(0.80-1.01)	6.7E-02	0.01	0.97	(0.75-1.26)	8.4E-01	0.27	1
	13q34	*F7*	rs6046	R353Q	112,821,160	A	0.11	1.00	(0.94-1.06)	9.8E-01	0.27	0.95	(0.89-1.02)	1.3E-01	1	1
	17p13.2	*GP1BA*	rs2243093	T-5C	4,776,675	C	0.13	1.03	(0.97-1.08)	3.6E-01	0.12	1.03	(0.94-1.13)	5.3E-01	1	2
	5q11.2	*ITGA2*	rs1062535[d]	T275T	52,387,170	A	0.39	1.01	(0.9701.05)	6.9E-01	0.36	0.99	(0.93-1.05)	6.7E-01	1	1
	17q21.32	*ITGB3*	rs5918	L33P	42,715,729	C	0.15	0.99	(0.94-1.05)	8.2E-01	0.09	0.89	(0.80-1.00)	4.0E-02	1	1
Metabolic																
	5q33.1	*ADRB2*	rs1042713	G16R	148,186,633	A	0.37	1.04	(1.00-1.08)	4.1E-02	0.44	0.99	(0.94-1.06)	8.7E-01	1	1
	5q33.1	*ADRB2*	rs1042714	E27Q	148,186,666	G	0.43	0.95	(0.91-0.98)	3.0E-03	0.22	1.03	(0.96-1.11)	3.9E-01	1	1
	5q33.1	*ADRB2*	rs1800888	I164T	148,187,078	T	0.01	0.94	(0.80-1.10)	4.4E-01	<0.01	-	-	-	0.16	1
	8p12	*ADRB3*	rs4994	W64R	37,942,955	G	0.07	0.99	(0.92-1.06)	6.7E-01	0.13	0.97	(0.89-1.06)	5.4E-01	0.43	1
	16q24.3	*CYBA*	rs4673	Y72H	87,240,737	A	0.34	1.04	(1.00-1.08)	6.9E-02	0.37	0.99	(0.93-1.05)	6.8E-01	1	1
	8q24.3	*CYP11B2*	rs1799998	T-344C	143,996,602	G	0.45	1.01	(0.97-1.04)	7.7E-01	0.44	0.99	(0.93-1.05)	7.0E-01	1	2
	10q23.33	*CYP2C9*	rs1057910	I359L	96,731,043	C	0.07	1.02	(0.95-1.10)	6.0E-01	0.11	0.99	(0.90-1.08)	7.7E-01	1	2
	10q23.33	*CYP2C9*	rs17443251[b]		96,697,880	C	0.13	1.02	(0.96-1.07)	5.2E-01	0.05	0.87	(0.76-0.99)	4.0E-02	1	2
	6q23.2	*ENPP1*	rs1044498	K121Q	132,214,061	C	0.14	1.03	(0.98-1.09)	2.8E-01	0.17	0.98	(0.91-1.06)	6.6E-01	1	2
	2q36.3	*IRS1*	rs1801278	G971R	227,368,788	T	0.07	1.09	(0.97-1.22)	1.4E-01	0.04	1.02	(0.83-1.25)	8.7E-01	0.20	1
	1p31.3	*LEPR*	rs1805096[e]	P1019P	65,874,845	A	0.38	0.99	(0.96-1.03)	7.2E-01	0.45	0.99	(0.93-1.05)	7.1E-01	1	1
	1p36.22	*MTHFR*	rs1801133	C677T	11,778,965	A	0.35	1.04	(1.00-1.08)	6.1E-02	0.16	1.04	(0.96-1.13)	2.9E-01	1	1
	1q43	*MTR*	rs1805087	D919G	235,115,123	G	0.19	1.01	(0.96-1.06)	7.7E-01	0.31	0.98	(0.91-1.04)	4.4E-01	1	2
	3p25.1-2	*PPARG*	rs1801282	Pro12Ala	12,368,125	G	0.12	1.01	(0.95-1.07)	7.2E-01	0.13	1.08	(1.00-1.18)	9.9E-02	1	1
	2p11.2	*VAMP8*	rs1010		85,662,493	C	0.42	1.06	(1.03-1.10)	8.7E-04	0.38	1.04	(0.97-1.10)	2.5E-01 #	1	2
Mineralcorticoid																
	17q23.3	*ACE*	rs4343[a]	Ins/Del	58,919,763	A	0.47	0.97	(0.93-1.01)	9.2E-02	0.41	1.05	(0.99-1.11)	1.3E-01	1	1
	1q42.2	*AGT*	rs699	M235T	228,912,417	G	0.41	1.03	(0.99-1.06)	1.9E-01	0.39	1.03	(0.97-1.10)	2.9E-01	1	1
	3q24	*AGTR1*	rs5186	A1166C	149,942,678	C	0.30	1.00	(0.96-1.04)	8.6E-01	0.09	1.01	(0.91-1.12)	8.0E-01	0.96	1
	14q32.3	*BDKRB2*	rs1799722	C-58T	95,740,892	T	0.49	0.99	(0.95-1.03)	6.3E-01	0.37	0.98	(0.92-1.04)	4.4E-01	0.18	2
Other																
	4p16.3	*ADD1*	rs4961	G460W	2,876,505	T	0.19	0.99	(0.95-1.04)	7.6E-01	0.17	1.02	(0.94-1.10)	6.8E-01	1	1
	9p21.3	*CDKN2A/B*	rs1333049		22,115,503	C	0.52	1.28	(1.23-1.33)	2.7E-34	0.51	1.12	(1.05-1.19)	3.3E-04	1	GWAS
	22q11.21	*COMT*	rs4680	V158M	18,331,271	G	0.48	0.99	(0.95-1.02)	5.2E-01	0.46	0.97	(0.91-1.03)	3.3E-01	1	2
	6q25.1	*ESR1*	rs2234693	T-401C	152,205,028	C	0.46	0.98	(0.95-1.02)	3.0E-01	0.42	1.02	(0.96-1.08)	5.6E-01	1	1
	6p22.1	*HFE*	rs1799945	H63D	26,199,158	G	0.15	0.97	(0.92-1.02)	2.4E-01	0.07	0.88	(0.79-0.99)	3.1E-02	1	2
	6p22.1	*HFE*	rs1800562	C282Y	26,201,120	A	0.06	1.03	(0.96-1.12)	3.9E-01	<0.01	-	-	-	1	2
	13q14.2	*HTR2A*	rs9526246[c]		46,347,862	A	0.42	1.05	(1.01-1.08)	1.9E-02	0.43	1.03	(0.97-1.10)	2.9E-01	1	2
	12p12.3	*MGP*	rs1049897[f]		14,925,788	A	0.39	1.03	(0.99-1.06)	1.9E-01	0.39	1.02	(0.96-1.08)	5.1E-01	1	2
	1q41	*MIA3*	rs17465637		220,890,152	C	0.73	1.08	(1.02-1.14)	3.8E-03	0.64	1.09	(1.00-1.18)	5.2E-02	1	GWAS
	1p36.22	*NPPA*	rs5065	R29X	11,906,068	G	0.15	0.97	(0.92-1.02)	2.9E-01	0.16	0.96	(0.89-1.04)	3.5E-01	1	1
	5q31.3	*NR3C1*	rs6195	N363S	142,759,510	C	0.03	0.96	(0.87-1.07)	4.8E-01	<0.01				1	2
	3q27.1	*THPO*	rs6141	A5713G	185,572,960	C	0.46	1.03	(0.99-1.07)	1.3E-01	0.47	0.97	(0.92-1.03)	3.6E-01	1	2

Variants ordered by biological pathway, then gene. Per-allele odds ratios are presented for the effect allele, which is the minor allele in European populations.

r^2 with best imputed proxy was estimated using ~2.5M directly genotyped or HapMap-imputed SNPs in the CARDIoGRAM Consortium.

Tagging levels are 1 (r^2>0.8 with all HapMap/Seattle SNPs of MAF≥0.02), 2 (r^2>0.5 with all HapMap/Seattle SNPs of MAF≥0.05), 3 (only non-synonymous and known functional variants of MAF>0.01) and GWAS (specific SNPs previously identified in recent GWAS).

[a] rs4343 has r^2=1 with the insertion/deletion polymorphism in the *ACE* gene in CEU HapMap 2 population.

[b] rs17443251 has r^2=0.75 with the more commonly studied R144C variant (rs1799853) in the *CYP2C9* gene in CEU HapMap 2 population.

[c] rs9526246 has r^2=0.97 with the more commonly studied T102C variant (rs6313) in the *HTR2A* gene in CEU HapMap 2 population.

[d] rs1062535 has r^2=0.97 with the more commonly studied C807T variant (rs1126643) in the *ITGA2* gene in CEU HapMap 2 population.

[e] rs1805096 has r^2=0.89 with the more commonly studied rs6700896 variant in the *LEPR* gene in CEU HapMap 2 population.

[f] rs1049897 has r^2=1 with the more commonly studied A102T variant (rs4236) in the *MGP* gene in CEU HapMap 2 population.

[g] rs4968624 has r^2=0.97 with the more commonly studied L125V variant (rs668) in the *PECAM1* gene in CEU HapMap 2 population.

[h] rs12944077 has r^2=1 with the more commonly studied S563N variant (rs12953) in the *PECAM1* gene in CEU HapMap 2 population.
