# Supplementary material for: Large-Scale Gene-Centric Analysis Identifies Novel Variants for Coronary Artery Disease
Source: PLoS Genet. 2011 Sep 22;7(9):e1002260. doi: 10.1371/journal.pgen.1002260 (PMC3178591; doi:10.1371/journal.pgen.1002260)
Supplement: Table S5 — 27 loci meeting P<1×10^−4^ threshold in discovery stage meta-analyses. Lead SNP = SNP with lowest P-value at this locus; risk allele = allele associated with increased CAD risk according to forward strand; freq = frequency of risk allele pooled across controls; OR = per-allele odds ratio for risk allele; 95% CI = 95% confidence interval around odds ratio; P = P value from fixed-effect meta-analysis; Combined = 10 European studies and 2 South Asian studies combined in a single fixed-effect meta-analysis; Overall = P values from discovery stage and replication stage combined; P_adj = P value adjusted for both study-specific and meta-analysis inflation factors in the discovery stage; SNPs ordered by ascending P value. * For 3 loci (ZC3HC1, CYP17A1, COL4A1/COL4A2), replication data are not presented here, however genome-wide significant associations at these loci are reported in the paper by the CARDIoGRAM Consortium. (PDF) [file pgen.1002260.s009.pdf]

Table S5. 27 loci meeting $P<10^{-4}$ threshold in discovery stage meta-analyses.

				risk	European				South Asian				Combined			Replication stage				Overall	
**Category**	**locus**	**lead SNP**	**nearest genes**	**allele**	**freq**	**OR**	**95% CI**	**P**	**freq**	**OR**	**95% CI**	**P**	**OR**	**95% CI**	**P**	**freq**	**OR**	**95% CI**	**P**	**P**	**P_adj**
**European**	20q11.22	rs867186	*MYH7B/PROCR/EDEM2*	A	0.90	1.18	(1.11-1.25)	1.9E-07	0.81	1.04	(0.96-1.12)	3.5E-01	1.12	(1.06-1.18)	3.5E-06	0.90	1.06	(1.01-1.12)	7.8E-03	3.2E-08	2.8E-07
	12p12.1	rs11047689	*BCAT1*	C	0.18	1.13	(1.07-1.19)	2.2E-06	0.20	0.92	(0.85-0.99)	2.4E-02	1.06	(1.01-1.10)	9.0E-03	0.19	1.00	(0.96-1.04)	4.9E-01	1.6E-05	8.6E-05
	10q23.31	rs2246942	*LIPA*	G	0.34	1.09	(1.05-1.14)	4.9E-06	0.49	1.07	(1.01-1.14)	2.8E-02	1.09	(1.05-1.12)	4.8E-07	0.36	1.07	(1.04-1.11)	3.8E-05	4.3E-09	2.5E-08
	7q32.2	rs11556924	*ZC3HC1*	C	0.62	1.09	(1.05-1.14)	7.6E-06	0.80	1.10	(1.02-1.18)	1.3E-02	1.09	(1.05-1.12)	3.1E-07			*		*	
	16q22.3	rs2000999	*HPR/HP/TXNL4B*	A	0.20	1.11	(1.06-1.16)	1.1E-05	0.37	1.05	(0.98-1.11)	1.5E-01	1.09	(1.05-1.13)	1.2E-05	0.20	1.03	(0.99-1.08)	4.7E-02	8.0E-06	3.7E-05
	10q24.32	rs3824755	*CYP17A1*	G	0.91	1.15	(1.08-1.22)	1.5E-05	0.76	1.12	(1.04-1.19)	1.8E-03	1.14	(1.09-1.19)	1.2E-07			*		*	
	4q12	rs3796529	*REST*	T	0.19	1.11	(1.06-1.17)	1.6E-05	0.23	1.05	(0.98-1.12)	1.9E-01	1.09	(1.05-1.14)	1.9E-05	0.19	1.02	(0.98-1.05)	2.2E-01	4.8E-05	2.0E-04
	8q24.13	rs17321515	*TRIB1*	A	0.53	1.08	(1.04-1.12)	2.2E-05	0.64	1.01	(0.95-1.08)	7.5E-01	1.06	(1.03-1.10)	1.3E-04	0.52	1.05	(1.02-1.08)	1.6E-03	6.5E-07	3.3E-06
	2p21	rs4299376	*ABCG8*	G	0.32	1.08	(1.04-1.13)	5.0E-05	0.27	1.03	(0.96-1.10)	3.6E-01	1.07	(1.04-1.11)	7.4E-05	0.29	1.05	(1.02-1.09)	1.7E-03	1.4E-06	6.0E-06
	13q34	rs4773144	*COL4A1/COL4A2*	G	0.44	1.08	(1.04-1.12)	5.5E-05	0.42	1.13	(1.06-1.20)	7.2E-05	1.09	(1.06-1.13)	3.5E-08			*		*	
	1q21.3	rs877343	*PMVK/ADAM15*	G	0.43	1.08	(1.04-1.12)	5.5E-05	0.62	1.02	(0.96-1.09)	4.5E-01	1.06	(1.03-1.10)	1.2E-04	0.41	1.02	(0.99-1.05)	1.2E-01	8.6E-05	3.6E-04
	15q26.1	rs2071410	*FURIN*	G	0.33	1.08	(1.04-1.12)	5.6E-05	0.24	1.06	(0.99-1.14)	7.5E-02	1.08	(1.04-1.12)	1.2E-05	0.33	1.05	(1.01-1.09)	8.0E-03	7.0E-06	2.8E-05
	17q11.2	rs4132610	*CDK5R1*	C	0.39	1.08	(1.04-1.12)	6.6E-05	0.48	1.02	(0.96-1.08)	5.3E-01	1.06	(1.03-1.10)	2.0E-04	0.39	1.02	(0.99-1.06)	7.0E-02	6.1E-05	2.2E-04
	17q23.1	rs1296279	*RPS6KB1*	A	0.19	1.10	(1.05-1.15)	7.1E-05	0.27	1.01	(0.94-1.08)	8.7E-01	1.07	(1.03-1.11)	7.5E-04	0.23	1.03	(0.99-1.06)	7.6E-02	7.1E-05	2.4E-04
	12q13.2	rs11171846	*TIMELESS*	T	0.09	1.13	(1.06-1.20)	7.8E-05	0.04	1.01	(0.87-1.17)	9.3E-01	1.11	(1.05-1.18)	2.3E-04	0.09	1.03	(0.96-1.11)	1.9E-01	1.8E-04	6.0E-04
**South Asian**	11p15.4	rs11041718	*TUB*	G	0.47	1.00	(0.97-1.04)	9.3E-01	0.43	1.15	(1.08-1.22)	1.1E-05	1.04	(1.01-1.07)	1.8E-02	0.47	1.01	(0.97-1.04)	3.8E-01	5.6E-05	1.2E-04
	6p21.33	rs3095235	*MICB (HLA region)*	C	0.83	1.04	(0.99-1.09)	1.5E-01	0.94	1.31	(1.16-1.49)	2.4E-05	1.07	(1.02-1.12)	4.1E-03	0.79	1.03	(0.99-1.07)	7.7E-02	2.6E-05	7.9E-05
	2q21.3	rs2322659	*LCT*	T	0.26	1.00	(0.95-1.06)	9.9E-01	0.51	1.13	(1.06-1.19)	9.8E-05	1.06	(1.01-1.10)	8.6E-03	0.21	1.00	(0.96-1.04)	5.5E-01	5.8E-04	2.0E-03
**Combined**	19p13.12	rs2074901	*CYP4F2*	C	0.17	1.09	(1.04-1.15)	3.4E-04	0.15	1.12	(1.03-1.22)	6.9E-03	1.10	(1.05-1.15)	8.3E-06	0.16	1.00	(0.96-1.04)	4.8E-01	5.3E-05	3.6E-04
	2p23.3	rs4665319	*HADHA*	C	0.79	1.09	(1.04-1.14)	2.6E-04	0.77	1.08	(1.01-1.16)	2.5E-02	1.09	(1.04-1.12)	1.8E-05	0.77	0.99	(0.96-1.03)	7.0E-01	1.5E-04	9.9E-04
	15q26.3	rs7173377	*IGF1R*	C	0.40	1.06	(1.02-1.10)	4.4E-03	0.58	1.11	(1.04-1.18)	7.8E-04	1.07	(1.04-1.11)	2.5E-05	0.38	0.98	(0.95-1.01)	9.2E-01	2.7E-04	1.3E-03
	7q34	rs2269997	*PARP12/TBXAS1*	G	0.79	1.09	(1.04-1.15)	1.0E-03	0.84	1.16	(1.04-1.28)	8.9E-03	1.10	(1.04-1.16)	4.1E-05	0.76	1.05	(1.01-1.09)	4.3E-03	2.9E-06	1.7E-05
	1q25.3	rs4631655	*C1orf21*	G	0.18	1.08	(1.03-1.13)	1.3E-03	0.17	1.11	(1.02-1.20)	1.2E-02	1.09	(1.04-1.13)	5.3E-05	0.20	1.00	(0.97-1.04)	4.6E-01	2.8E-04	1.3E-03
	3q25.2	rs1371097	*P2RY1*	T	0.15	1.07	(1.02-1.13)	5.5E-03	0.20	1.12	(1.04-1.21)	2.6E-03	1.09	(1.04-1.14)	6.6E-05	0.16	0.98	(0.94-1.02)	8.7E-01	6.2E-04	2.6E-03
	1q32.1	rs868407	*TNNT2*	C	0.28	1.07	(1.03-1.11)	1.0E-03	0.18	1.10	(1.01-1.19)	2.0E-02	1.08	(1.04-1.11)	6.7E-05	0.33	1.01	(0.98-1.05)	2.1E-01	1.7E-04	6.6E-04
	6p25.3	rs2569881	*FOXC1*	G	0.86	1.11	(1.05-1.18)	1.2E-04	0.94	1.07	(0.94-1.22)	2.8E-01	1.11	(1.05-1.16)	7.1E-05	0.88	1.03	(0.98-1.08)	1.0E-01	9.1E-05	3.6E-04
	5q31.1	rs2706399	*IL5*	G	0.51	1.07	(1.03-1.11)	4.3E-04	0.46	1.06	(1.00-1.12)	7.1E-02	1.07	(1.03-1.10)	8.0E-05	0.51	1.05	(1.02-1.09)	1.5E-03	2.1E-06	8.9E-06

lead SNP = SNP with lowest P-value at this locus; risk allele = allele associated with increased CAD risk according to forward strand; freq = frequency of risk allele pooled across controls; OR = per-allele odds ratio for risk allele; 95% CI = 95% confidence interval around odds ratio; P = P value from fixed-effect meta-analysis; Combined = 10 European studies and 2 South Asian studies combined in a single fixed-effect meta-analysis; Overall = P values from discovery stage and replication stage combined; P_adj = P value adjusted for both study-specific and meta-analysis inflation factors in the discovery stage;

SNPs ordered by ascending P value.

* For 3 loci (*ZC3HC1*, *CYP17A1*, *COL4A1/COL4A2*), replication data are not presented here, however genome-wide significant associations at these loci are reported in the paper by the CARDIoGRAM Consortium.
