# Supplementary material for: Large-Scale Gene-Centric Analysis Identifies Novel Variants for Coronary Artery Disease
Source: PLoS Genet. 2011 Sep 22;7(9):e1002260. doi: 10.1371/journal.pgen.1002260 (PMC3178591; doi:10.1371/journal.pgen.1002260)
Supplement: Table S6 — Details of studies included in the replication stage. All values are means (±SD) unless otherwise stated. (PDF) [file pgen.1002260.s010.pdf]

Table S6. Details of studies included in the replication stage.

	CARDIoGRAM Consortium									
**Study**	**ADVANCE**	**deCODE**	**GerMIFS I**	**GerMIFS III**	**GerMIFS III**	**CADomics**	**MedStar**	**MIGen**	**OHGS**	**EPIC-NL**
Cases / Controls	278 / 312	6640 / 27,611	884 / 1604	1222 / 1287	1157 / 1748	2078 / 2952	874 / 447	1274 / 1407	1542 / 1455	1172 / 1650
Age at diagnosis (yrs)	45.8 (6.2)	74.8 (11.8)	50.2 (7.8)	51.4 (7.5)	58.6 (8.7)	60.8 (10.1)	48.9 (6.4)	42.4 (6.6)	48.7 (7.3)	60.6 (8.4)
Male (%)	41.6	50.9	49.9	59.3	65.5	63.8	56.2	61.5	64	30.6
MI (n, %)	140 (50.4)	3632 (54.7)	884 (100)	1222 (100)	1157 (100)	1211 (58.3)	420 (48.1)	1274 (100)	950 (61.6)	341 (30.3)

All values are means (±SD) unless otherwise stated.
