# Supplementary material for: Large-Scale Gene-Centric Analysis Identifies Novel Variants for Coronary Artery Disease
Source: PLoS Genet. 2011 Sep 22;7(9):e1002260. doi: 10.1371/journal.pgen.1002260 (PMC3178591; doi:10.1371/journal.pgen.1002260)
Supplement: Table S7 — Expression QTL (eQTL) analysis for novel CAD loci. a. eQTL analysis for novel CAD loci. †Key (Proportion of all Probes). 1 = Weak (0%–20%). 2 = Medium (20%–80%). 3 = Strong (80%–100%). b. Conditional analysis of expression QTL (eQTL) loci. Conditional analysis of the lead LIPA SNP on a secondary SNP at the same locus that is also associated with gene expression shows that the lead SNP at the LIPA locus has a strong independent effect on LIPA expression levels. Conditional analysis of the lead IL5 SNP on a second nearby SNP that is also associated with RAD50 gene expression shows that the observed eQTL association with the IL5 SNP is probably due to LD with the RAD50 SNP. (PDF) [file pgen.1002260.s011.pdf]

Table S7a. Expression QTL (eQTL) analysis for novel CAD loci.

Region (lead SNP)	Gene	Gene expressed?	Lead SNP monocyte association P-value	Strength[†]
LIPA (rs2246942)	*LIPK*	NO		
	LIPN	N/A		
	LIPM	N/A		
	ANKRD22		0.6426	2
	STAMBPL1		0.1400	2
	ACTA2		0.0043	2
	FAS		0.0089	2
	CH25H	NO		
	LIPA		1.01E-124	3
	IFIT2		0.7876	3
	IFIT3		0.9306	2
	IFIT1L	NO		
	IFIT1		0.6344	2
	IFIT5		0.3111	2
	SLC16A12		0.9830	3
	PANK1		0.9559	1
IL5 (rs2706399)	*IL3*	NO		
	CSF2	NO		
	PDLIM4	NO		
	P4HA2		0.9575	1
	SLC22A4		0.0518	2
	SLC22A5		0.7884	2
	LOC441108	NO		
	IRF1		0.1019	3
	IL5	NO		
	RAD50		6.65E-06	2
	IL13	NO		
	IL4	NO		
	KIF3A	NO		
	CCNI2	N/A		
	SEPT8	N/A		
	ANKRD43	NO		
	SHROOM1		0.0194	1
	GDF9	NO		
	UQCRQ		0.2126	3
	LEAP2		0.1657	2
	AFF4		0.8323	2
	ZCCHC10	NO		
TRIB1 (rs17321515)	*ZNF572*	NO		
	SQLE		0.9429	2
	KIAA0196		0.6054	3
	NSMCE2		0.9458	2
	TRIB1		0.9259	3
ABCG8 (rs4299376)	*THADA*		0.5084	2
	LOC728819	N/A		
	PLEKHH2	NO		
	DYNC2LI1		0.3672	2
	ABCG5	NO		
	ABCG8	NO		
	LRPPRC		0.8337	2
	PPM1B		0.8186	1
	SLC3A1	NO		
	PREPL		0.5951	2

[†]Key (Proportion of all Probes)
1 = Weak (0-20%)
2 = Medium (20-80%)
3 = Strong (80-100%)

Table S7b. Conditional analysis of expression QTL (eQTL) loci.

Gene	Lead SNP	Lead SNP monocyte association P-value	Strength†	Secondary SNP used for conditional analysis (r^2 with lead SNP)	Secondary SNP monocyte association P-value	P-value of lead SNP when conditioned on secondary SNP	Independent eQTL signal?	Proxy for secondary SNP in CAD meta-analysis (r^2)	P value for CAD association with proxy SNP
LIPA	rs2246942	1.01E-124	3	rs2250781 (0.492)	1.54E-96	1.63E-46	Yes	rs2250781 (1)	2.31E-05
RAD50	rs2706399	6.65E-06	2	rs17772583 (0.181)	2.58E-23	0.6698	No	rs2069812 (0.759)	0.20

Conditional analysis of the lead *LIPA* SNP on a secondary SNP at the same locus that is also associated with gene expression shows that the lead SNP at the *LIPA* locus has a strong independent effect on *LIPA* expression levels.
Conditional analysis of the lead *IL5* SNP on a second nearby SNP that is also associated with *RAD50* gene expression shows that the observed eQTL association with the *IL5* SNP is probably due to LD with the *RAD50* SNP.
