# Supplementary material for: Large-Scale Gene-Centric Analysis Identifies Novel Variants for Coronary Artery Disease
Source: PLoS Genet. 2011 Sep 22;7(9):e1002260. doi: 10.1371/journal.pgen.1002260 (PMC3178591; doi:10.1371/journal.pgen.1002260)
Supplement: Table S8 — Comparison of haplotype frequencies for novel loci in European and South Asian controls. Haplotypes are displayed in decreasing frequency, with the same haplotype order in both ethnicities. * = haplotype frequencies in bold are those containing the CAD risk-associated allele of the lead SNP. SNPs were selected for inclusion in the haplotype if they had r2≥0.5 in either the European or the South Asian controls. The 3330 PROCARDIS controls were used to represent the European populations, whilst the PROMIS controls were used to represent the South Asian population. Only haplotypes that were common (frequency>5%) in at least one population are displayed. (PDF) [file pgen.1002260.s012.pdf]

Table S8. Comparison of haplotype frequencies for novel loci in European and South Asian controls.

Locus	Lead SNP	SNPs in haplotype	Europeans		South Asians	
			SNP frequency	Haplotype frequencies*	SNP frequency	Haplotype frequencies
LIPA	rs2246942	3	0.35	0.66/**0.29**/**0.04**	0.50	0.48/**0.41**/**0.11**
TRIB1	rs17321515	2	0.46	0.53/**0.47**	0.35	0.63/**0.37**
ABCG8	rs4299376	1	0.33	N/A	0.28	N/A
IL5	rs2706399	2	0.48	0.50/**0.43**/**0.08**	0.53	0.44/**0.43**/**0.13**

Haplotypes are displayed in decreasing frequency, with the same haplotype order in both ethnicities.

* = haplotype frequencies in bold are those containing the CAD risk-associated allele of the lead SNP

SNPs were selected for inclusion in the haplotype if they had $r^2 \geq 0.5$ in either the European or the South Asian controls. The 3330 PROCARDIS controls were used to represent the European populations, whilst the PROMIS controls were used to represent the South Asian population.

Only haplotypes that were common (frequency>5%) in at least one population are displayed.
