# Supplementary material for: Large-Scale Gene-Centric Analysis Identifies Novel Variants for Coronary Artery Disease
Source: PLoS Genet. 2011 Sep 22;7(9):e1002260. doi: 10.1371/journal.pgen.1002260 (PMC3178591; doi:10.1371/journal.pgen.1002260)
Supplement: Text S1 — Supplementary Methods, Supplementary References, Supplementary Acknowledgements. (PDF) [file pgen.1002260.s013.pdf]

Large-Scale Gene-Centric Analysis Identifies Novel Variants

for Coronary Artery Disease

The IBC 50K CAD Consortium

TEXT S1

SUPPLEMENTARY MATERIALS AND METHODS **pg2**

SUPPLEMENTARY REFERENCES **pg10**

SUPPLEMENTARY ACKNOWLEDGEMENTS **pg13**

SUPPLEMENTARY MATERIALS AND METHODS

DISCOVERY STAGE STUDIES

10 studies of Europeans (11,202 CAD cases / 30,733 controls) were included in the discovery stage, as were 2 South Asian studies (4394 cases / 4259 controls). Further statistics relating to these studies are presented in **Table 1**/**Table S1**, and descriptions of each of the studies are given below.

Atherosclerosis Risk in Communities (ARIC)
The ARIC study is a prospective population-based study of atherosclerosis and cardiovascular diseases in 15,792 men and women, including 11,478 non-Hispanic whites and 4,314 African-Americans, drawn from 4 U.S. communities (suburban Minneapolis, Minnesota; Washington County, Maryland; Forsyth County, North Carolina, and Jackson, Mississippi) [1]. Prevalent CHD was defined as self-reported myocardial infarction or revascularization procedures. Coronary heart disease cases were defined as participants with: (1) myocardial infarction, (2) heart surgery, (3) coronary bypass, (4) angioplasty of coronary artery, or (5) physician-diagnosed history of myocardial infarction. Those patients with angina only were excluded from the coronary heart disease cases. The ARIC study participated in this project as part of the National Heart, Lung, and Blood Institute's (NHLBI's) CARe Consortium [2].

BLOODOMICS - Dutch
The Dutch component of the BLOODOMICS collaboration comprises of cases drawn from the Academic Medical Centre Amsterdam Premature Atherosclerosis Study (AMC-PAS) and the AGNES study, with controls ascertained from the Sanquin Common Controls (SANQUIN-CC) study.

AMC-PAS/Sanquin: Cases were recruited as part of a prospective cohort study (AMC-PAS) with symptomatic CAD before the age of 51 years, defined as MI, coronary revascularization, or evidence of at least 70% stenosis in a major epicardial artery. A collection of DNA samples from blood donors from the north-west region of the Netherlands was established as controls for this study. Participating donors were recruited at routine Sanquin Blood Bank donation sessions (SANQUIN-CC). More than 95% of the controls are from the same region as the cases of the AMC-PAS cohort.

AGNES: The AGNES case-control set consisted of individuals with a first acute ST-elevation myocardial infarction [3], hence the whole set was considered as cases in this project. AGNES cases had ECG-registered ventricular fibrillation occurring before reperfusion therapy for an acute and first ST-elevation myocardial infarction. AGNES controls were individuals with a first acute ST-elevation myocardial infarction but without ventricular fibrillation. All cases and controls were recruited at seven heart centers in The Netherlands from 2001–2008. Individuals with an actual non-ST-elevation myocardial infarction, prior myocardial infarction, congenital heart defects, known structural heart disease, severe comorbidity, electrolyte disturbances, trauma at presentation, recent surgery, previous coronary artery bypass graft or use of class I and III antiarrhythmic drugs were excluded. Individuals who developed ventricular fibrillation during or after percutaneous coronary intervention were not eligible. Furthermore, because early reperfusion limits the opportunity of developing ventricular fibrillation, potential control subjects undergoing percutaneous coronary intervention within 2h after onset of myocardial ischemia symptoms were not included. This time interval was based on the observation that >90% of cases developed ventricular fibrillation within 2h after onset of symptoms.

BLOODOMICS - German
The German component of the BLOODOMICS collaboration comprises of cases and controls from the LUdwigshafen RIsk and Cardiovascular Health (LURIC) study, supplemented by additional controls from the Mannheim study.

LURIC: LURIC is a prospective study of cardiovascular death in individuals of German ancestry resident in southwest Germany who underwent elective coronary angiography and left ventriculography between June 1997 and January 2000 [4]. CHD in the current analyses was defined by troponin confirmed MI (ie, acute ST or non-ST- elevation MI or based on past medical records) or presence of visible luminal narrowing of ≥50% in at least one coronary vessel. Individuals with ≥20% but <50% stenosis were excluded from the analyses. Individuals with stenosis <20% were regarded as controls. Fasting blood samples collected before angiography were kept frozen at –80°C between the day of blood draw and the day of analysis for total cholesterol, HDL-C and triglycerides (all determined enzymatically).

Mannheim: Additional controls consisted of 1187 healthy, unrelated blood donors between 18–68 years of age [5]. They were recruited in 2004 and 2005 by the Institute of Transfusion Medicine and Immunology (Mannheim, Germany) and share the ethnic background with the LURIC patients. According to the German guidelines for blood donation, all blood donors were examined by a standard questionnaire. All blood donors consented to the use of their samples for research studies.

British Heart Foundation Family Heart Study (BHF-FHS)
Cases were European Caucasians who had a validated history of either myocardial infarction (MI) or coronary revascularisation (coronary artery bypass surgery or percutaneous coronary angioplasty) before their 66th birthday [6]. Recruitment was carried out on a national basis in the UK through (i) responses to a sustained UK-wide media campaign (ii) through responses to posters placed within hospitals and GP (family physician) surgeries throughout the UK and (iii) in a pilot-phase contacting patients listed on computer based coronary artery disease databases in the two lead centres (Leeds and Leicester). The recruitment period was from April 1998 to November 2003. Controls consisted of European Caucasian blood donors between 30-70 years of age recruited throughout the UK through the UK National Blood Service as part of the Wellcome Trust Case Control Consortium study [7]. Apart from age and gender, limited information was available on the controls.

Cardiovascular Health Study (CHS)
The Cardiovascular Health Study (CHS) is a population-based, longitudinal study of coronary heart disease and stroke in adults aged 65 years and older [8]. The main objective of the study is to identify factors related to the onset and course of coronary heart disease and stroke. CHS is designed to determine the importance of conventional cardiovascular disease (CVD) risk factors in older adults, and to identify new risk factors in this age group, especially those that may be protective and modifiable. The study design called for enrolment of 1250 men and women in each of four communities: Forsyth County, North Carolina; Sacramento County, California; Washington County, Maryland; and Pittsburgh, Pennsylvania. Eligible participants were sampled from Medicare eligibility lists in each area. Extensive physical and laboratory evaluations were performed at baseline to identify the presence and severity of CVD risk factors such as hypertension, hypercholesterolemia and glucose intolerance; subclinical disease such as carotid artery atherosclerosis and ventricular enlargement; and clinically overt CVD. Coronary heart disease cases were defined as participants with: (1) myocardial infarction, (2) heart surgery, (3) coronary bypass, (4) angioplasty of coronary artery, or (5) physician-diagnosed history of myocardial infarction. Those patients with angina only were excluded from the coronary heart disease cases. The CHS participated in this project as part of the NHLBI's CARe Consortium [2].

Coronary Artery Risk Development in Young Adults (CARDIA)
The CARDIA study is a prospective, multi-center investigation of the natural history and etiology of cardiovascular disease in African Americans and whites 18-30 years of age at the time of initial examination [9]. The initial examination included 5,115 participants selectively recruited to represent proportionate racial, gender, age, and education groups from four communities: Birmingham, AL; Chicago, IL; Minneapolis, MN; and Oakland, CA. Participants from the Birmingham, Chicago, and Minneapolis centers were recruited from the total community or from selected census tracts. Participants from the Oakland center were randomly recruited from the Kaiser-Permanente health plan membership. Five sequential examinations have been conducted from the time of initiation of the study in 1985 to 1986 through year 15 (2000 to 2001). Coronary heart disease cases were defined as participants with: (1) myocardial infarction, (2) heart surgery, (3) coronary bypass, (4) angioplasty of coronary artery, or (5) physician-diagnosed history of myocardial infarction. Those patients with angina only were excluded from the coronary heart disease cases. The CARDIA study participated in this project as part of the NHLBI's CARe Consortium [2].

Framingham Heart Study (FHS)
The Framingham Heart Study is a longitudinal observational, community based cohort initiated in 1948 in Framingham, Massachusetts, to prospectively investigate CVD and its risk factors. The children (and spouses of the children) of the original cohort, labeled the Offspring cohort, were recruited in 1971, and have been examined approximately every 4 years since [10]. At each clinic examination, participants receive routine questionnaires, a physical examination, anthropometry, electrocardiograms, and blood tests. At the second (1978-1982), fourth (1987-1990), fifth (1991-1995), and sixth (1996-1998) examination cycles participants underwent transthoracic echocardiography. Coronary heart disease cases were defined as participants with: (1) myocardial infarction, (2) heart surgery, (3) coronary bypass, (4) angioplasty of coronary artery, or (5) physician-diagnosed history of myocardial infarction. Those patients with angina only were excluded from the coronary heart disease cases. Participants included in the current study were drawn from the Framingham Offspring cohort and the Third Generation Cohort. The FHS participated in this project as part of the NHLBI's CARe Consortium [2].

The London Life Sciences Prospective Population Cohort (LOLIPOP)
LOLIPOP is an ongoing population based cohort study of ~30,000 Indian Asian and European white men and women, aged 35-75 years, recruited from the lists of 58 General Practitioners in West London, United Kingdom [11]. Response rates have averaged 62%; there are no material differences between responders and non-responders with respect to age, sex, co-morbidity and available risk factors. For the present study, DNA was available for 4929 Indian Asian participants. Indian Asians were selected if all four grandparents originated from the Indian subcontinent.

MONICA-KORA
The MONItoring of trends and determinants in CArdiovascular disease/Cooperative Health Research in the Region of Augsburg (MONICA/KORA) Augsburg study consists of a series of independent population-based epidemiological surveys of participants living in the region of Augsburg, Southern Germany initially conducted to estimate the prevalence and distribution of cardiovascular risk factors among individuals aged 25 to 74 years as part of the World Health Organization MONICA project [12]. All survey participants were residents of German nationality identified through the registration office and were examined in 1984/85 (S1), 1989/90 (S2) and 1994/95 (S3). Among a source population of 9,300 participants aged 35 to 74 years without prevalent CHD and with available blood samples a case-cohort design was performed including a stratified random sample of 2,163 participants and all CHD cases in the source population [13]. All participants underwent standardized examinations including blood withdrawals for plasma and DNA [14]. Genotypes obtained using the HumanCVD beadchip (Illumina, CA) were available on up to 1,688 MONICA/KORA

participants of the case-cohort study with 275 CHD cases and 1,413 CHD non-cases excluding subjects with individual SNP call rate < 95%. The mean follow-time was 10.5 years (standard deviation 4.8). CHD was defined as incident fatal or non-fatal myocardial infarction or sudden cardiac death occurring before the age of 75 years and was identified through the MONICA/KORA Augsburg coronary event registry and through follow-up questionnaires for subjects who had moved out of the study area. For deceased subjects information on causes of death was obtained.

PennCATH

The Penn-CATH cohort is a University of Pennsylvania (U. Penn) Medical Center based coronary angiographic study that has been used previously for replication of novel genes and risk factors for atherosclerotic CVD and type-2 diabetes [15,16]. Briefly, PennCATH, recruited between July 1998 and March 2003, provides an ongoing focus for analyzing the association of biochemical and genetic factors with coronary atherosclerosis in a consecutive cohort of patients undergoing cardiac catheterization and coronary angiography at Penn. A total of 3,850 subjects provided written informed consent in a Penn Institutional Review Board approved protocol. Enrolment criteria included any clinical indication for cardiac catheterization and ability to give informed consent. The following data were extracted from the medical record; age, gender, self-reported race/ethnicity, past medical (including diabetes, hypertension, dyslipidemia, prior MI and cardiac events), social, family and medication history, cardiovascular risk factors, physical exam including vital signs, weight and height (for BMI). Ethnicity information was self-reported. Coronary angiograms were scored at the time of procedure by the interventional cardiologist. Blood was drawn in a fasting state, DNA (buffy coats) and plasma was isolated, and lipoproteins and glucose were assayed on all samples.

A nested case-control candidate-gene study was performed in PennCATH (N=1,596 Caucasians) composed of controls (N=512) who on coronary angiography showed no or minimal (<10% stenosis of any vessel) evidence of CAD and angiographic CAD) and cases (N=1086) with one or more coronary vessels with ≥50% stenosis. Controls were aged over 40 in men and women. Cases were selected to be young ≤70.

Precocious Coronary Artery Disease (PROCARDIS)

Ascertainment criteria for PROCARDIS probands were MI or symptomatic acute coronary syndrome (SACS), on the assumption that the latter represents a similar pathological process according to modified World Health Organisation diagnostic criteria before the age of 66 y [17]. Diagnosis of MI required documentation of two or more of: (a) typical ischemic chest pain, pulmonary oedema, syncope or shock; (b) development of pathological Q-waves and/or appearance or disappearance of localized ST-elevation followed by T-wave inversion in two or more standard electrocardiograph leads; (c) increase in concentration of serum enzymes consistent with MI (e.g. creatine kinase more than twice the upper limit of normal). Diagnosis of SACS required documentation of hospitalization for one of the following indications: (a) unstable angina diagnosed by typical ischemic chest pain at rest associated with reversible ST-depression in two or more standard electrocardiograph leads; (b) thrombolysis for suspected MI (as indicated by localized ST-elevation in two or more standard electrocardiograph leads) even without later development of T-wave inversion, Q-waves, or a significant enzyme rise; or (c) emergency revascularization (i.e. during same admission) following presentation with typical ischemic chest pain at rest. Probands completed questionnaires in order to recruit affected siblings with a range of CAD diagnoses at age < 66 y (MI, SACS, chronic stable angina, or intervention for coronary revascularization), who were then invited to participate in the study if their diagnoses were confirmed. Parents and up to four unaffected siblings per family were recruited wherever possible to augment the recovery of linkage phase information. Families were recruited in four countries: in Sweden and the UK, recruitment was based on existing databases of patients hospitalized for CAD; in Sweden for a few families and in Germany for all, recruitment was through lists of MI patients currently or recently admitted to coronary care units (CCUs); in Italy, recruitment

was through both MI patient databases and CCUs. 99.5% of the study participants reported having a white European ancestry.

Pakistan Risk of Myocardial Infarction Study (PROMIS)
PROMIS is an ongoing case-control study of acute myocardial infarction (MI) in six centres in urban Pakistan [18]. Participants in the present study were recruited between 2005 and 2008. MI cases had symptoms within 24 hours of hospital presentation, typical electrocardiographic changes, and a positive troponin-I test. Controls were individuals without a history of cardiovascular disease. They were frequency-matched to cases by sex and age (in 5 year bands) and concurrently identified in the same hospitals as index cases because they were either: (i) visitors of patients attending the outpatient department; (ii) patients attending the outpatient department for routine noncardiac complaints, or (iii) nonblood related visitors of index MI cases. People with recent illnesses or infections were not eligible. Information was recorded on personal and paternal ethnicity, spoken language, dietary intake, lifestyle factors and other characteristics.

REPLICATION STAGE STUDIES

Individuals included in the replication stage were predominantly from the CARDIoGRAM Consortium (where they had not already been included in the discovery stage of this study). Data provided from the CARDIoGRAM Consortium on the SNPs to be tested for replication were either measured or imputed from GWA assays. An additional study, the EPIC-NL study, also provided data from a HumanCVD Beadchip array. These studies comprised 17,121 coronary disease cases and 40,473 controls in total (**Table S6**).

CARDIoGRAM Consortium
Basic characteristics of the CARDIoGRAM studies used in the replication stage are shown in **Table S6**. Detailed information about these studies can be found in [19].

EPIC-NL
The EPIC-NL cohort is the Dutch contribution to the European Prospective Investigation into Cancer and Nutrition, recruited between 1993 and 1997, and consists of the Prospect cohort, a prospective population based cohort of 17,357 women between 49-70 years, participating in breast cancer screening, and the Monitoring Project on Risk Factors for Chronic Diseases (MORGEN) cohort, consisting of 22,654 men and women between 20-59 years at recruitment in three Dutch towns (Amsterdam, Maastricht and Doetinchem). At baseline, a general questionnaire containing questions on demographic characteristics, smoking, presence of chronic diseases and other potential risk factors was filled out by all participants. Body weight, height, waist, and hip circumference were also measured, and a non-fasting blood sample was taken. Information on incident coronary heart disease occurrence during follow-up was obtained through linkage with the database of hospital discharge diagnoses from the Dutch National Medical Registry. Mortality information was obtained through linkage with the Cause of Death Registry at Statistics Netherlands. CHD cases were persons with a first event (either fatal or non-fatal of ICD-9:410-414, incl. sub codes 427.5, 798.1, 798.2, 798.9 and ICD-10:I20, I23 t/m I25, incl. all sub codes) [20]. 95.5% of the participants is Caucasian, based on MDS clustering.

STATISTICAL ANALYSES

Simulation study

To determine suitable P value thresholds for taking SNPs forward from the discovery stage to the replication stage and for determining 'chip-wide significance', controls from the BHF-FHS study were used to conduct a simulation. Firstly, rare (MAF <1%) SNPs were removed, as were individuals with >5% genotype missingness, leaving 31,490 SNPs in total. For each chromosome in turn, two random groups of 250 participants were drawn and Cochran-Armitage trend tests were performed for each SNP. To check that the size of sample drawn did not affect the simulations, we repeated the simulations with two random groups of 500 participants and found identical results. For each of 1000 such simulations, the smallest P value across the chromosome and the number of SNPs passing certain thresholds (eg, $P<10^{-3}$, $P<10^{-4}$, $P<10^{-5}$) were counted. 50,000 simulations were then conducted taking a random value from the 1000 available for each chromosome and calculating the smallest P value across the whole array and the number of SNPs exceeding each threshold across the whole array. A P value threshold of $P<10^{-4}$ was selected for taking SNPs forward for replication based on the expected number of significant SNPs under the null hypothesis (**Figure S1a**). The median number of significant SNPs was 2 (mean 2.5), suggesting that using this threshold for taking SNPs to the replication stage would be likely to result in few false positives. The comparable numbers for a threshold of $P<10^{-3}$ are median=27 (mean 27), whilst the mean was 0.25 for $P<10^{-5}$. A threshold of $P<3x10^{-6}$ for 'chip-wide significance' was selected based on the 5th percentile of the minimum P values seen, equating to a chip-wide alpha of 0.05 (**Figure S1b**).

Association analyses and Quality Control

Each of the 12 discovery stage studies (15,596 CAD cases / 34,992 controls) conducted independent association tests on their own participants. In each study, individuals were excluded if they were cryptically related, drawn from specific groups (eg, familial hyperlipidemia patients), or had low genotyping call rates (<95%). SNPs were excluded from analyses if they: had a low (<98%) genotyping call rate; violated Hardy-Weinberg equilibrium in the controls (p<0.0001); were monomorphic; or were located on the sex chromosomes. Unadjusted genotypic association tests using a case-control design were conducted using PLINK for all studies with the exception of PROCARDIS and FHS (where adjustments for relatedness were made due to the family-based study designs), PROMIS (where adjustment for the first 4 prinicipal components was made to reduce overdispersion by accounting for population substructure), and MONICA-KORA (where a case-cohort design was employed, adjusting for age, sex and date of survey using the SAS programme PHREG). Attempts were also made to reduce overdispersion in LOLIPOP through adjustment for principal components and removal of ethnic outliers, but this did not affect the inflation factor. Genomic inflation factors, estimated as the ratio of the median observed chi^2 statistic to the median expected chi^2 statistic, were less than 1.10 for all studies with the exception of LOLIPOP (inflation factor = 1.145, **Table S2**). The German MI Family Study was excluded from the analyses of the European discovery stage studies due to its high level of overdispersion (inflation factor = 1.331) and concerns about differential missingness for some SNPs.

Combined analyses and Quality Control

Results from all SNPs from all of the discovery stage studies were collated and all SNPs were aligned to the forward strand according to NCBI Strand 36. SNPs were removed from individual studies if the study-specific control-based P value for HWE was less than $1x10^{-4}$, the MAF was 0, if the MAF was unusually discrepant, or if no association result was available. SNPs were removed from all studies if they were non-autosomal, or if the pooled ethnic-specific MAF in controls was less than 1%. In total, results from 36,779 SNPs were available for combining.

The primary combined analysis was a fixed-effect inverse-variance weighted meta-analysis performed separately for each ethnic group using STATA v11. An additional analysis combined European and South Asian studies to identify additional variants common to both ethnicities. Manhattan plots for the ethnic-specific and combined meta-analyses are shown in **Figure 2**. Power to detect associated variants was estimated for the discovery stage meta-analysis of the European studies, indicating that for variants with MAF of 5%, we had 90% power to detect a per-allele odds ratio of 1.05 (**Figure S1a**).

Heterogeneity was assessed using the Q statistic and quantified using the I^2 statistic [21]. A chi-squared test for between-ethnicity heterogeneity was also performed. Sensitivity analyses were conducted using a random-effects meta-analysis as well as a fixed-effect meta-analysis which accounted for overdispersion in the individual studies (standard errors were multiplied by the square root of the study's inflation factor) and overdispersion/between-study heterogeneity in each meta-analysis (chi^2 values divided by the square root of the meta-analysis-specific P value). Novel SNPs that passed the threshold to be taken forward for replication ($P<10^{-4}$) were grouped into loci with the lead variant from each locus being taken forward. 27 SNPs were selected for replication (**Table S5**), however 3 were later excluded as they were in loci that were recently discovered by the CARDIoGRAM GWA Consortium (*COL4A1/COL4A2, ZC3HC1, CYP17A1*) and were therefore no longer considered novel.

Replication studies

Data for the 24 SNPs to be tested for replication were obtained from non-overlapping participants from the CARDIoGRAM GWA Consortium and the EPIC-NL study. (The Utrecht Cardiovascular Pharmacogenetics study also shared data on these SNPs from the HumanCVD Beadchip array, however these data were excluded from the replication analysis as the controls were hypertensive individuals). Odds ratios and standard errors from both measured and imputed SNPs were combined across replication studies for the relevant SNPs using a fixed-effect meta-analysis. Independent replication was deemed to have occurred if the one-tailed P value was less than a 0.05 threshold after Bonferroni adjustment for the number of tests (n=27, $P=1.9\times10^{-3}$). Two-tailed P values discovery stage and one-tailed P values from the replication stage were combined using Fisher's method with a chip-wide P value of 3×10^{-6} considered to be statistically significant based on the simulation study.

Further statistical analyses

4 novel SNPs showed evidence of independent replication, so additional analyses were performed on these regions. Forest plots for the discovery stage studies are shown in **Figure S3**. To check for consistency of effect, subgroup analyses were performed in the replication stage studies for MI cases only, CAD cases aged less than 50, males only and females only (**Figure S4**). Haplotype frequencies including the lead SNP from each replicated locus were compared between Europeans and South Asians using controls from the PROCARDIS and PROMIS studies respectively. All SNPs with $r^2\geq0.5$ in either population were included in the haplotypes and the frequencies of all common (frequency>5%) haplotypes were compared across the control populations (**Table S8**).

Heritability estimates were calculated locus-by-locus using the multifactorial liability threshold model [22] based on odds ratio estimates that assume that the lead SNP at a locus accurately tags the disease-causing variant (spreadsheet used for calculation available from authors on request). The estimates were uncorrected for potential amplification due to the "winners-curse" or to attenuation due to clinically unscreened control data. The variance was then summed across the loci assuming that they have independent effects. The calculations are based on a disease prevalence estimate of 5% (www.heartstats.org) and an estimate of 40% for the total heritability of coronary disease which takes into account that our case series were a mix of men and women who had all

survived coronary disease [23,24]. Taking all of these issues into account, we estimate that ~9% of the total heritability of coronary disease can be explained by the 34 common variants (**Figure 7**).

Other CVD risk factors

Association results for the 4 novel coronary disease-associated SNPs were looked up in previous meta-analyses of GWA studies of known cardiovascular risk factors: systolic blood pressure [25], LDL- and HDL-cholesterol [26], triglycerides [26] and type 2 diabetes [27]. Effect estimates, standard errors and P values were estimated according to the statistical analyses conducted in each study.

Expression QTL (eQTL) analyses

eQTL analyses related to the 4 novel coronary disease-associated loci were carried out in monocyte transcriptomes from 395 healthy blood donors (recruited from one centre) and 363 patients with premature myocardial infarction (recruited from 4 centres) assembled by the Cardiogenics consortium (http://www.cardiogenics.eu) [28]. All subjects were of European Caucasian origin. RNA was extracted from monocytes isolated from whole blood with CD14 micro beads (AutoMacs Pro, Miltenyi). Genomic DNA was extracted from peripheral blood by standard procedures. Gene expression profiling was performed using Illumina Human Ref-8 arrays (Illumina Inc., San Diego, CA) containing 24,516 probes. mRNA was amplified and labelled using the Illumina Total Prep RNA Amplification Kit (Ambion, Inc., Austin, TX). After hybridization, array images were scanned using the 7 Illumina BeadArray Reader and probe intensities were extracted using the Gene expression module of the Illumina's Bead Studio software. Variance Stabilization Transformation (VST) was applied to the raw intensities and quantile normalization was performed in the R statistical environment using the Lumi and beadarray packages. Whole-genome genotyping was carried out using either the Human Custom 1.2M or the Human Quad Custom 670 arrays from Illumina. The expression level of all genes within 1 Mb of each lead SNP was first assessed. For those genes showing expression, an indication of the relative level of expression (low, medium or high) was assigned as within the top 20%, the bottom 20% or between 20-80% of genes expressed for that gene in monocytes. The associations of lead SNPs with transcript levels of expressed genes at individual loci were assessed using additive regression models adjusted for age, gender and centre using Stata software (n=758). Proxy SNPs (r^2>0.9) were used (if available) if the lead SNP was not available on the genotyping platform. Where a lead SNP showed a significant association with expression, the region was assessed for additional SNPs also showing an eQTL effect on the relevant gene. If another SNP was found showing a stronger association with expression while in weak linkage with the lead SNP (r^2<0.5), a conditional analysis was carried out to determine if the association of the lead SNP with expression was independent of the other variant. The full findings from this analysis are shown in **Table S7a/b**. The principal finding was the strong independent association of the lead SNP at the *LIPA* locus with mRNA levels for *LIPA* (**Figure 6/Table S7**). The association was seen independently in the blood donors (P=2.5×10^{-62}) and in the coronary cases (P=3.5×10^{-65}). An association was also seen between the lead SNP at the *IL5* locus with mRNA levels of neighbouring gene *RAD50*, however this association was completely attenuated when conditioned on the lead SNP in the *RAD50* gene driving this association. This suggests that the lead SNP at *IL5* has no eQTL effect, although *IL5* was not sufficiently expressed in monocytes or macrophages to be analysed.

SUPPLEMENTARY REFERENCES

1. The ARIC Investigators (1989) The Atherosclerosis Risk in Communities (ARIC) Study: design and objectives. *Am J Epidemiol* 129: 687-702.

2. Musunuru K, Lettre G, Young T, Farlow DN, Pirrucello JP *et al*. (2010) Candidate gene association resource (CARe): design, methods, and proof of concept. *Circ Cardiovasc Genet* 3: 267-275.

3. Bezzina CR, Pazoki R, Bardai A, Marsman RF, de Jong JS *et al*. (2010) Genome-wide association study identifies a susceptibility locus at 21q21 for ventricular fibrillation in acute myocardial infarction. *Nat Genet* 42: 688-691.

4. Winkelmann BR, Marz W, Boehm BO, Zotz R, Hager J *et al*. (2001) Rationale and design of the LURIC study--a resource for functional genomics, pharmacogenomics and long-term prognosis of cardiovascular disease. *Pharmacogenomics* 2: S1-73.

5. Bugert P, Hoffmann MM, Winkelmann BR, Vosberg M, Jahn J *et al*. (2003) The variable number of tandem repeat polymorphism in the P-selectin glycoprotein ligand-1 gene is not associated with coronary heart disease. *J Mol Med* 81: 495-501.

6. Samani NJ, Burton P, Mangino M, Ball SG, Balmforth AJ *et al*. (2005) A genomewide linkage study of 1,933 families affected by premature coronary artery disease: The British Heart Foundation (BHF) Family Heart Study. *Am J Hum Genet* 77: 1011-1020.

7. Wellcome Trust Case Control Consortium (2007) Genome-wide association study of 14,000 cases of seven common diseases and 3,000 shared controls. *Nature* 447: 661-678.

8. Fried LP, Borhani NO, Enright P, Furberg CD, Gardin JM *et al*. (1991) The Cardiovascular Health Study: design and rationale. *Ann Epidemiol* 1: 263-276.

9. Friedman GD, Cutter GR, Donahue RP, Hughes GH, Hulley SB *et al*. (1988) CARDIA: study design, recruitment, and some characteristics of the examined subjects. *J Clin Epidemiol* 41: 1105-1116.

10. Feinleib M, Kannel WB, Garrison RJ, McNamara PM, Castelli WP (1975) The Framingham Offspring Study. Design and preliminary data. *Prev Med* 4: 518-525.

11. Chambers JC, Elliott P, Zabaneh D, Zhang W, Li Y *et al*. (2008) Common genetic variation near MC4R is associated with waist circumference and insulin resistance. *Nat Genet* 40: 716-718.

12. Hense HW, Filipiak B, Doering A, Stieber J, Liese A, Keil U (1998) Ten-year trends of cardiovascular risk factors in the MONICA Augsburg region in southern Germany: results from 1984/1985, 1989/1990, and 1994/1995 surveys. *CVD Prev* 1: 318-327.

13. Koenig W, Khuseyinova N, Baumert J, Thorand B, Loewel H *et al*. (2006) Increased concentrations of C-reactive protein and IL-6 but not IL-18 are independently associated with incident coronary events in middle-aged men and women: results from the MONICA/KORA Augsburg case-cohort study, 1984-2002. *Arterioscler Thromb Vasc Biol* 26: 2745-2751.

14. Wichmann HE, Gieger C, Illig T (2005) KORA-gen--resource for population genetics, controls and a broad spectrum of disease phenotypes. *Gesundheitswesen* 67: S26-S30.

15. Lehrke M, Millington SC, Lefterova M, Cumaranatunge RG, Szapary P *et al*. (2007) CXCL16 is a marker of inflammation, atherosclerosis, and acute coronary syndromes in humans. *J Am Coll Cardiol* 49: 442-449.

16. Kathiresan S, Voight BF, Purcell S, Musunuru K, Ardissino D *et al*. (2009) Genome-wide association of early-onset myocardial infarction with single nucleotide polymorphisms and copy number variants. *Nat Genet* 41: 334-341.

17. Clarke R, Peden JF, Hopewell JC, Kyriakou T, Goel A *et al*. (2009) Genetic variants associated with Lp(a) lipoprotein level and coronary disease. *N Engl J Med* 361: 2518-2528.

18. Saleheen D, Zaidi M, Rasheed A, Ahmad U, Hakeem A *et al*. (2009) The Pakistan Risk of Myocardial Infarction Study: a resource for the study of genetic, lifestyle and other determinants of myocardial infarction in South Asia. *Eur J Epidemiol* 24: 329-338.

19. Preuss M, König IR, Thompson JR, Erdmann J, Absher D *et al.* (2010) Design of the Coronary ARtery DIsease Genome-Wide Replication And Meta-Analysis (CARDIoGRAM) Study -- A Genome-wide association meta-analysis involving more than 22,000 cases and 60,000 controls. *Circ Cardiovasc Genet* 3: 475-483.

20. Beulens JW, Monninkhof EM, Verschuren WM, van der Schouw YT, Smit J *et al*. (2010) Cohort Profile: The EPIC-NL study. *Int J Epidemiol* 39: 1170-1178.

21. Higgins JP, Thompson SG, Deeks JJ, Altman DG (2003) Measuring inconsistency in meta-analyses. *BMJ* 327: 557-560.

22. Falconer DS (1965) The inheritance of liability to certain diseases,estimated from the incidence among relatives. *Ann Hum Genet* 29: 51-76.

23. Zdravkovic S, Wienke A, Pedersen NL, Marenberg ME, Yashin AI, de Faire U (2002) Heritability of death from coronary heart disease: a 36-year follow-up of 20 966 Swedish twins. *J Intern Med* 252: 247-254.

24. Zdravkovic S, Wienke A, Pedersen NL, de Faire U (2007) Genetic influences on angina

pectoris and its impact on coronary heart disease. *Eur J Hum Genet* 15: 872-877.

25. Newton-Cheh C, Johnson T, Gateva V, Tobin MD, Bochud M *et al*. (2009) Genome-wide association study identifies eight loci associated with blood pressure. *Nat Genet* 41: 666-676.

26. Teslovich TM, Musunuru K, Smith AV, Edmondson AC, Stylianou IM *et al*. (2010) Biological, clinical and population relevance of 95 loci for blood lipids. *Nature* 466: 707-713.

27. Zeggini E, Scott LJ, Saxena R, Voight BF, Marchini JL *et al*. (2008) Meta-analysis of genome-wide association data and large-scale replication identifies additional susceptibility loci for type 2 diabetes. *Nat Genet* 40: 638-645.

28. Heinig M, Petretto E, Wallace C, Bottolo L, Rotival M *et al*. (2010) A trans-acting locus regulates an anti-viral expression network and type 1 diabetes risk. *Nature* 467: 460-464.

SUPPLEMENTARY ACKNOWLEDGEMENTS

CARe Consortium: The following nine parent studies have contributed parent study data, ancillary study data, and DNA samples through the Broad Institute of MIT and Harvard (N01-HC-65226) to create this genotype/phenotype database for wide dissemination to the biomedical research community: Atherosclerotic Risk in Communities (ARIC): University of North Carolina at Chapel Hill (N01-HC-55015, N01-HC-55018), Baylor Medical College (N01-HC-55016), University of Mississippi Medical Center (N01-HC-55021),University of Minnesota (N01-HC-55019), Johns Hopkins University (N01-HC-55020), University of Texas, Houston (N01-HC-55022); Cardiovascular Health Study (CHS): University of Washington (N01-HC-85079, N01-HC-55222, U01-HL-080295), Wake Forest University (N01-HC-85080), Johns Hopkins University (N01-HC-85081, N01-HC-15103), University of Pittsburgh (N01-HC-85082), University of California, Davis (N01-HC-85083), University of California, Irvine (N01-HC-85084), New England Medical Center (N01-HC- 85085), University of Vermont (N01-HC-85086), Georgetown University (N01-HC-35129), University of Wisconsin (N01-HC-75150); Cleveland Family Study (CFS): Case Western Reserve University (R01-HL-46380, M01-RR-00080); Cooperative Study of Sickle Cell Disease (CSSCD): University of Illinois (N01-HB-72982, N01-HB-97062), Howard University (N01-HB-72991, N01-HB-97061), University of Miami (N01-HB-72992, N01-HB-97064), Duke University (N01-HB-72993), George Washington University (N01-HB-72994), University of Tennessee (N01-HB-72995, N01-HB-97070), Yale University (N01-HB-72996, N01-HB- 97072), Children's Hospital-Philadelphia (N01-HB-72997, N01-HB-97056), University of Chicago (N01-HB-72998, N01-HB-97053), Medical College of Georgia (N01-HB-73000, N01- HB-97060), Washington University (N01-HB-73001, N01-HB-97071), Jewish Hospital and Medical Center of Brooklyn (N01-HB-73002), Trustees of Health and Hospitals of the City of Boston, Inc., (N01-HB-73003), Children's Hospital-Oakland (N01-HB-73004, N01-HB-97054), University of Mississippi (N01-HB-73005), St. Luke's Hospital-New York (N01-HB-73006), Alta Bates-Herrick Hospital (N01-HB-97051), Columbia University (N01-HB-97058), St. Jude's Children's Research Hospital (N01-HB-97066), Research Foundation, State University of New York-Albany (N01-HB-97068, N01-HB-97069), New England Research Institute (N01-HB- 97073), Interfaith Medical Center-Brooklyn (N01-HB-97085); Coronary Artery Risk in Young Adults (CARDIA): University of Alabama at Birmingham (N01-HC-48047, N01-HC-95095), University of Minnesota (N01-HC-48048), Northwestern University (N01-HC-48049), Kaiser Foundation Research Institute (N01-HC-48050), Tufts-New England Medical Center (N01-HC- 45204), Wake Forest University (N01-HC-45205), Harbor-UCLA Research and Education Institute (N01-HC-05187), University of California, Irvine (N01-HC-45134, N01-HC-95100); Framingham Heart Study (FHS): Boston University (N01-HC-25195, R01-HL-092577, R01- HL-076784, R01-AG-028321); Jackson Heart Study (JHS): Jackson State University (N01- HC-95170), University of Mississippi (N01-HC-95171), Tougaloo College (N01-HC-95172); Multi-Ethnic Study of Atherosclerosis (MESA): University of Washington (N01-HC-95159), University of California, Los Angeles (N01-HC-95160), Columbia University (N01-HC-95161), Johns Hopkins University (N01-HC-95162, N01-HC-95168), University of Minnesota (N01-HC- 95163), Northwestern University (N01-HC-95164), Wake Forest University (N01-HC-95165), University of Vermont (N01-HC-95166), New England Medical Center (N01-HC-95167), Harbor-UCLA Research and Education Institute (N01-HC-95169), Cedars-Sinai Medical Center (R01-HL-071205), University of Virginia (subcontract to R01-HL-071205); Sleep Heart Health Study (SHHS): Johns Hopkins University (U01-HL-064360), Case Western University (U01- HL-063463), University of California, Davis (U01-HL-053916), University of Arizona (U01- HL-053938, U01-HL-053934), University of Pittsburgh (U01-HL-077813), Boston University (U01-HL-053941), MedStar Research Institute (U01-HL-063429), Johns Hopkins University (U01-HL-053937). CARe was performed with the support of the National Heart, Lung, and Blood Institute and acknowledges the contributions of the research institutions, study investigators, and field staff in creating this resource for biomedical research.
